# A Systematic Review of Aircraft Disinsection Efficacy

**DOI:** 10.3390/insects16090911

**Published:** 2025-09-01

**Authors:** Gregory Hawley, Michael Klowak, Syed Zain Ahmad, Candice Madakadze, Jahmar Hewitt, Aquilla Reid-John, Asal Adawi, Andrea K. Boggild

**Affiliations:** 1 Department of Medicine, University of Toronto, Toronto, ON M5G 1G6, Canada; 2 Tropical Disease Unit, Toronto General Hospital, University Health Network, Toronto, ON M5G 2C4, Canada; 3 Institute of Medical Science, University of Toronto, Toronto, ON M5S 3H2, Canada; 4 Department of Physiology, University of Toronto, Toronto, ON M5S 3H2, Canada; 5 Temerty Faculty of Medicine, University of Toronto, Toronto, ON M5S 3H2, Canada

**Keywords:** *Aedes*, aircraft, *Anopheles*, arthropod vector, *Culex*, disinsection, insecticide, marine vessel, mosquito, vector-borne disease

## Abstract

Disinsection of aircraft and other conveyances is recommended to prevent the international spread of disease-carrying mosquitoes. A systematic review synthesized the effectiveness of adult mosquito disinsection aboard international air, marine, and land conveyances, using literature available up to 31 May 2025. Nineteen experimental trials, nine of which included an unexposed control arm, were synthesized. The studies were generally of poor quality with high risk of bias, and adherence to WHO guidelines was 33.30% (range: 18.20–60.5%). Across comparator trials of aircraft disinsection, the odds of mosquito mortality in the treated groups compared to control groups was 163.60 (95% CI 147–182), and the relative risk of mosquito death was 14.24 (95% CI 12.99–15.63). The direction of effect was consistent across mosquito species, methods of disinsection, types of aircraft, and insecticides, though the magnitude of the effect varied widely. The only WHO-recommended insecticide tested in a controlled trial was 2% d-phenothrin, which demonstrated an odds ratio of 171.70 (95% CI 139.10–212) and a relative risk of 20.08 (95% CI 16.53–24.43) for mosquito mortality. The lack of adherence to WHO guidelines raises uncertainty about the real-world effectiveness of disinsection.

## Simple Summary

Disinsection, or treating conveyances such as airplanes, ships, and land transport with insecticides, is important for stopping the spread of mosquitoes that transmit diseases, including malaria and dengue fever. Preventing the global movement of these vectors helps reduce the spread of such diseases. A systematic review was conducted to assess how effective disinsection is at killing adult mosquitoes on international aircraft, marine vessels, and land transports. This review included 19 studies that tested insecticide treatments for mosquitoes, but most studies were of poor quality and carried a high risk of bias. Only one third of studies on aircraft disinsection followed guidelines set by the World Health Organization (WHO). The studies showed that disinsection significantly increased mosquito deaths, but the effectiveness varied widely depending on the type of insecticide, method, and mode of transport. Only one WHO-recommended insecticide (2% d-phenothrin) was tested in comparator trials, which demonstrated strong mosquito-killing results. The findings suggest that while disinsection can be effective, the lack of standardized studies makes it difficult to assess how well it works in real-world settings.

## 1. Introduction

Insect vectors of infectious disease agents may be introduced to locations in which they were not previously present via aircraft and other means of international transit [1]. Moreover, insects (vectors) may transmit pathogens to people in places serviced by aircraft (such as exported mosquitoes that transmit “airport malaria”). The World Health Organization (WHO) has formally published guidance on aircraft disinsection, initially in 1995 in the International Programme of Chemical Safety series [2], with reiteration of the importance of disinsection of aircraft departing from airports in disease-endemic areas in 2000 [3]. The International Health Regulations (2005) (IHR) [4] set out requirements for countries to develop capacities to prevent, protect against, control, and provide a public health response to the international spread of disease, including vector-borne diseases, in ways that are proportional to the public health risks. The IHR (2005) defines disinsection as “the procedure whereby health measures are taken to control or kill the insect vectors of human disease present in baggage, cargo, containers, conveyances, goods and postal parcels (IHR, Part I, article 1)” and states that it should “be carried out so as to avoid injury and, as far as possible, discomfort to persons” (IHR, Part IV, article 22, Section 3) [4]. A list of countries with specific aircraft disinsection regulations can be found in Table S1.

In addition, the IHR Annex 5 states that State Parties shall establish vector surveillance and control programmes at designated Points of Entry, extending at least 400 m from areas used for operations involving travellers, conveyances, containers, cargo, and postal parcels [4]. Consistent with this requirement, the International Civil Aviation Organization (ICAO), which harmonizes standards in civil aviation, including aircraft and at airports, encourages Member States to complete the Airport Vector Control Register maintained by ICAO while emphasizing the importance of guidance on vector surveillance and control in airports [5]. In 2016, an expert group was convened by WHO in response to the spread of Zika virus, which concluded that disinsection would have little effect in preventing importation of the virus, as it is imported mainly by infected travellers and, to a lesser extent, by mosquito vectors [6]. Thereafter, a WHO consultation in 2018 recognized that guidance on aircraft disinsection methods and procedures was required, with standard operating procedures for aircraft disinsection and training materials and tools [7]. The first edition of the WHO aircraft disinsection methods and procedures was published in 2021 [8] and then updated in 2023 [1]. Among other things, the latest guidance provides updates on methods of insecticide application and necessary associated equipment (Table S2), aerosol and residual spray specifications, and updated protocols for pre-embarkation and pre-departure cabin treatment.

Vehicular conveyances, including all forms of marine, rail, ground, and aircraft transport, contribute to the global spread of infectious diseases such as dengue [9], chikungunya [10], Western equine encephalitis (WEE) [11], Zika [12,13], and malaria [14] via movement of infected people as well as transmission-capable adult vectors. Consequently, vehicular disinsection via chemical insecticidal agents, including aerosol sprays, has been indiscriminately utilized to eliminate relevant vectors, including mosquitoes. There has been a demonstrable increase and geographical spread of mosquito borne infections in recent years. However, the evidence review of the effectiveness of disinsection of aircraft was last published in 2020, which captured literature only up to 31 December 2018 [15]. As such, an urgent update of the evidence review to inform the decision-making process of stakeholders under the current situation of intense dengue transmission and spread of invasive mosquito vector species was warranted, the results of which are presented herein. Additionally, although the risk of the introduction of vectors, including disease agents, to locations in which they were not previously present exists by other modes of transport, including land and water (such as sea, lake, and river) as well, an evidence review for non-aviation modes of transport has never been done. As such, the scope of the systematic review reported herein encompasses non-aviation modes of transport as well. A list of countries with specific marine conveyance disinsection regulations can be found in Table S3.

## 2. Materials and Methods

**Figure 1:**
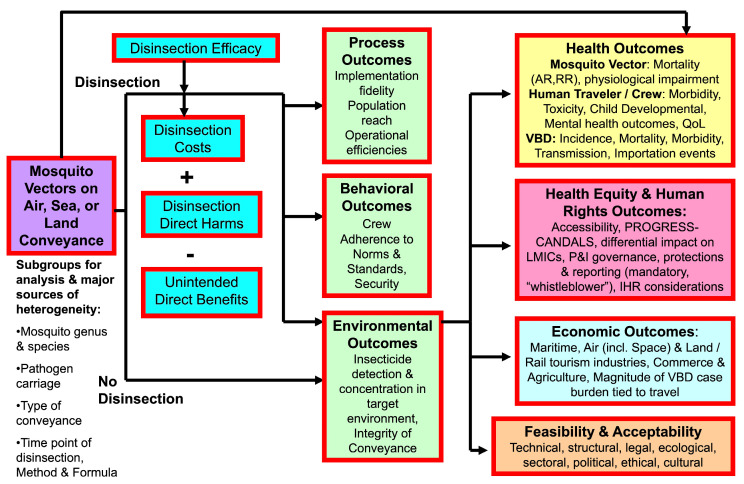
Analytic framework mapping the population of interest to intermediate and ultimate outcomes.

A systematic review was commissioned by the WHO [16] and conducted in accordance with the analytic framework outlined in Figure 1.

Though the body of work was not intended to be a guideline development, the process described in the WHO Guideline development handbook was followed for quality control. This systematic review synthesizes the effectiveness of disinsection of international travel carriers (passenger chamber, cargo area, cargoes, and air-cans) of all modes of transportation (air, water, and land transport) to prevent or reduce the spread of mosquito vectors via international travel (Table 1).

The systematic review was commissioned according to the population, intervention, comparator, and outcome (PICO) framework above. The systematic review was conducted according to the Preferred Reporting Items for Systematic Reviews and Meta-Analysis (PRISMA) guidelines [17] and was registered in the International Prospective Register of Systematic Reviews, PROSPERO (CRD42024543998).

**Table 1:** Population, intervention, comparator, and outcome for the effectiveness of disinsection.

**Question:** What is the effectiveness of disinsection of international travel carriers versus no disinsection is (passenger chamber, cargo area, cargoes, air-cans or containers) of all modes of transportation (air, water, and land transport) to prevent or reduce the spread of mosquito vectors via international travel?	
**Population**	Mosquitoes (by species)
**Intervention**	Disinsection of international travel carriers (passenger chamber, cargo area, cargoes, air-cans, or containers) of all modes of transportation (air, water, land, and transport) (by chemical [18] or non-chemical agent, method used, and other).
**Comparator**	No disinsection of international travel carriers (passenger chamber, cargo area, cargoes, air-cans, or containers) of all modes of transportation (air, water, and land transport)
**Outcome**	(i) No or reduced number of mosquitoes on aircrafts (passenger chamber, flight deck, cargo area, air-cans, or containers) and cargoes (water or land transport)

### 2.1. Inclusion Criteria

We included all papers relating to disinsection of international travel carriers (passenger chamber, cargo area, cargoes, and air-cans) of all modes of transportation (air, water, land, and transport) by chemical agent (including but not limited to DDT, d-phenothrin, and permethrin), method used, and targeting mosquitoes. Systematic reviews, randomized controlled trials (RCTs), cohort studies, cross-sectional studies, case-control studies, case-series, and case reports (n ≥ 1) were all included. Studies with alternative methodological designs but reporting primary data (e.g., conference presentations) were also included.

### 2.2. Exclusion Criteria

We excluded studies reporting only in vitro data, animal studies, and those which did not permit assessment of any pre-determined outcomes of interest (e.g., mathematical modeling studies). We further excluded studies conducted in putative models of conveyances that did not fully replicate the conveyance environment (e.g., non-pressurized shed as a model of an aircraft cabin; pieces of carpet treated with insecticide in a laboratory as a model of residual disinsection, etc.).

### 2.3. Outcomes

Outcomes of interest are as described above for the PICO framework (Table 1). Likewise, the broader analytic framework (Figure 1) encompasses multiple outcome domains, including unintended human health and safety effects of disinsection, the prevalence of mosquitoes aboard international conveyances, the prevalence of mosquitoes at ports of entry (including airport malaria), secondary operational public health, and systems-level outcomes. These additional outcome domains will be addressed in subsequent systematic reviews. The primary outcome addressed herein and leading to the generation of the Summary of Findings tables reported here is the efficacy of disinsection, with the outcomes of relative and absolute mosquito mortality.

### 2.4. Search Strategy

Six electronic databases (PubMed, Embase, Medline, Scopus, LILACS, and CINAHL) were searched from inception to 31 May 2025 without language restriction using the following search terms: (disinsection OR insecticide OR d-phenothrin OR permethrin OR deet OR spraying OR “mosquito control”) AND (travel OR airport OR airplane OR plane OR aviation OR aircraft OR airline OR air-cans OR truck OR bus OR cargo OR rail OR train OR tram OR marine OR ship OR boat OR lorry OR vessel OR submarine OR space OR spacecraft OR rocketship OR spaceship OR “marine vehicle” OR “marine vessel” OR “cruise ship” OR “water taxi” OR ferry OR barge OR “passenger chamber” OR “cargo area” OR “land transport vehicles”) AND (neurotoxicity OR crew OR passengers OR “flight attendant” OR “occupational exposure” OR insect OR mosquito OR malaria OR “airport malaria” OR dengue OR chikungunya OR zika).

**Figure 2:**
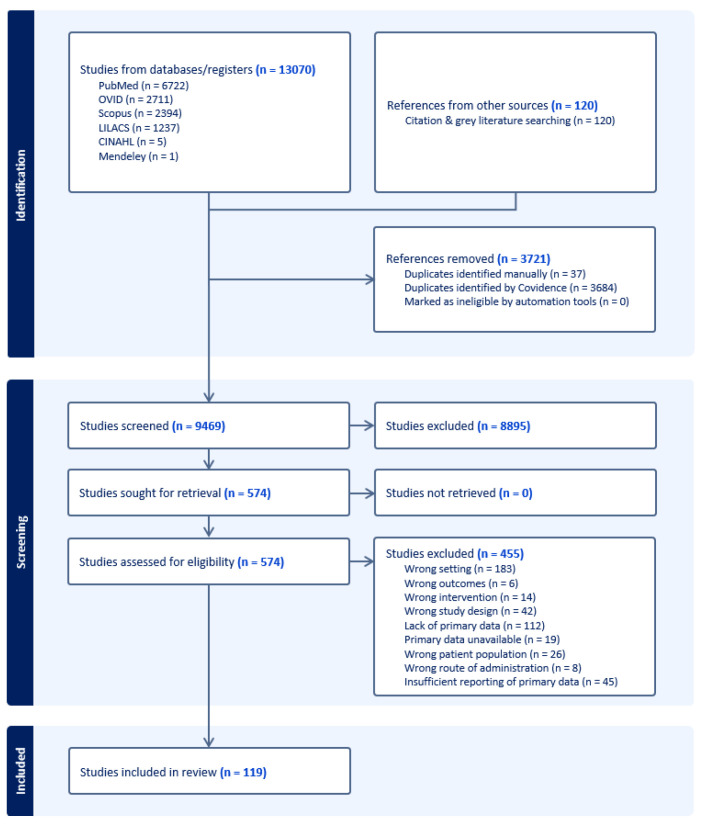
PRISMA flow diagram of literature evaluated for inclusion in systematic review of mosquito disinsection of international conveyances and surveillance of mosquitoes aboard conveyances and at points of entry.

OpenGrey and the Grey Literature Report databases were also searched for additional literature, including conference proceedings, dissertations, and other documents. Bibliographies of key papers were also hand-searched for relevant literature not captured by the above search strategy. Following de-duplication, this strategy identified a total of 9469 unique studies, which underwent double-screening by two authors at the title and abstract stage (Figure 2). After title and abstract screening, 574 unique papers were identified for full-text screening (Figure 2). Following double-review by two authors, 455 full-texts were excluded for reasons indicated in the PRISMA flow diagram (Figure 2) and 119 were included for data extraction and synthesis across all outcomes of interest.

Document organization and deduplication, as well as title, abstract, and full-text screening, were executed using the online platform Covidence. Articles were independently double-screened by two reviewers and any discrepancies were resolved through discussion and, in the event of non-agreement, by a tertiary arbitrator.

Data extraction was conducted by two independent reviewers and verified and collated by the study lead according to the Grading of Recommendations, Assessment, Development, and Evaluation (GRADE) framework [19,20,21]. Non-English articles were screened and extracted by native speaking reviewers or were translated into English using Google Translate (Google, Mountain View, CA, USA) in their absence. Non-English language full-texts provided as image files via interlibrary loan were converted to PDF and then run through optical character recognition software [22,23,24] in order to facilitate Google translation. All discrepancies were resolved through discussion between reviewers and disagreements were arbitrated by a third reviewer. Following extraction, data were represented in Characteristics tables and then synthesized in aggregate quantitatively and qualitatively, and “Summary of Findings” tables, where applicable, were generated using GRADEpro GDT (Cochrane, Oxford, UK). Where outcomes were reported inconsistently or different types of data were collected and reported, narrative synthesis was completed.

### 2.5. Data Analysis

Continuous variables were collected and reported as sample sizes, means with standard deviations, mean differences, and medians with ranges and interquartile ranges where applicable. Dichotomous or categorical variables (e.g., presence of mosquito) were collected and reported as frequencies and proportions with 95% confidence intervals when provided. Continuous outcomes (mean difference) and dichotomous outcomes (relative risk and odds ratio) were collected when available and reported in Summary of Findings tables, only when the primary study included a comparator group, using a standardized measure of treatment difference. Odds ratios reflect the odds of mosquito death in the experimental (exposed) arms compared to the odds of mosquito death in the control (unexposed) study arms, and were used to generate Forest plots using PRISM v9.0 (GraphPad, La Jolla, CA, USA). Similarly, relative risk reflects the risk of mosquito death in the experimental (exposed) arms compared to the risk of mosquito death in the control (unexposed) arms. Summary estimates of both continuous and dichotomous outcomes were pooled for each combination of disinsection (across methods, insecticide formulations, conveyance settings, and participants) and efficacy. Level of significance was set at a 5% alpha level for summary estimates of outcomes measured against a comparator. Statistical analysis was carried out using GRADEpro GDT (McMaster University, 2014), PRISM v.9.0 (GraphPad, La Jolla, CA, USA), and Review Manager (RevMan, computer program, version 5.3. Copenhagen: The Nordic Cochrane Centre, The Cochrane Collaboration, 2014).

### 2.6. Risk of Bias and Certainty of Evidence

Comprehensive risk of bias (ROB) forms adapted from the Joanna Briggs critical appraisal tools were designed and subsequently utilized independently and simultaneously by two reviewers to carry out the bias assessment [25]. The GRADE framework was followed to assess methodological quality, assigning each included study a quality grade of high, moderate, low, or very low, based on the apparent level of bias [19,20]. Discrepancies were resolved through discussion and, in the case of non-agreement, by a tertiary arbitrator. If outcomes were not reported or incompletely reported, studies were considered at risk for reporting and/or information/outcome bias, respectively. Bias assessments were pooled and an overall ROB score was achieved per study. A pooled assessment of bias risk was assigned based on the adequacy or inadequacy of—where applicable—allocation, concealment, blinding, attrition, and completeness of reporting by one reviewer and verified by a second reviewer. Study quality was reported using heatmaps generated by the software RevMan.

Where relevant, additional GRADE parameters such as inconsistency, indirectness, and imprecision of outcomes, as well as levels of publication bias and plausible confounding, effect size, and relevant dose response gradients, were also considered when grading certainty of evidence. Certainty was planned to be upgraded by one additional unit if a large effect size (<0.5 or >2) was evident or by two additional units if a very large effect size (<0.2 or >5) was reported. Overall, ROB was then considered alongside these additional GRADE parameters to generate a final certainty of evidence GRADE score, per reported outcome.

For the primary outcome—efficacy of disinsection as measured by mosquito mortality—many of the quality assessment elements of GRADE are not applicable, as the study participants were mosquitoes and the desired outcome was mortality. As such, the elements of participant blinding, loss to follow-up, and participant non-adherence to the intervention are not applicable. As such, a novel quality assessment checklist (Table S4) was developed by four study authors based on the WHO’s published “Guidelines for testing the efficacy of insecticide products used in aircraft” [26], which enabled study authors to score each disinsection trial across multiple domains of study design and implementation in order to achieve a composite grade of methodological rigor. The checklist contains 5 major sections scoring studies across 36 domains of methodological quality in accordance with the guidelines. If studies adhered to a particular recommendation, they received 1 point for that domain. If studies did not adhere to a particular recommendation, they received no points for that domain. If partial adherence occurred, then studies received 0.5 points for that domain. As not all domains were relevant to all study designs, studies were scored only on the domains to which they could have theoretically adhered, and then assigned a percentage adherence score. For example, a disinsection study trialing an insecticide at “blocks away” would not have been scored on the domains only relevant to pre-embarkation disinsection procedures.

Where common outcomes (e.g., insecticidal efficacy) were reported by more than one study of similar design (e.g., use of same insecticide in similar setting), forest plots were generated for pooled odds ratios of mosquito mortality collectively and across subgroups including mosquito species, model of conveyance, method of disinsection, and type of insecticide using GraphPad PRISM v.9.0 (GraphPad, La Jolla, CA, USA).

Sources of anticipated effect heterogeneity that would influence the efficacy outcome, in particular, encountered in this systematic review include but are not limited to the genus (e.g., *Aedes* vs. *Anopheles*) and species (e.g., *Aedes aegypti* Linnaeus vs. *Aedes albopictus* Skuse) of mosquito; pathogen-carriage vs. non-carriage (e.g., DENV, CHIKV, or ZIKV detected in mosquito vector or not); type, formulation, and concentration of insecticide applied (e.g., 2% permethrin vs. 2% d-phenothrin); air filtration system operational at the time of insecticide application and number of air exchanges per unit time on conveyance; model of conveyance to which insecticide was applied (e.g., Airbus vs. Boeing models of aircraft); climatologic factors including ambient temperature, humidity, UV exposure, cabin pressure, and altitude at which insecticide was applied; and the point in time of travel at which the insecticide was applied (e.g., pre-embarkation vs. time of descent). The process of disinsection is, by nature, multistep with variability inherent to persons applying the insecticide, and as such would be considered a “complex intervention”. Additionally, the participants to which disinsection was applied (i.e., mosquitoes) are highly likely to be variably located around the space to which the intervention is applied in real life (i.e., stationary and hidden or enclosed vs. airborne) and may be of different species with highly variable tolerabilities to insecticides, and as such would themselves introduce complexity. Insecticides are unlikely to be applied uniformly by persons of variable height, strength, and stride cadence. Dispersion of insecticide is likely to be affected by the aforementioned climatologic and ambient cabin factors, including the operation of the air conditioner, as is recommended [26]. All such factors were collected in as granular a manner as was reported by the primary study and summarized in the Study Characteristics tables. An absence of this degree of granularity was noted in the limitations of data generalizability and applicability section of the descriptive text.

## 3. Results

### 3.1. Literature Search

Of 9469 unique studies identified by our search, 574 proceeded to full-text screening, at which point 455 were excluded for failing to fulfill inclusion criteria (Figure 2). Of 119 unique included reports or studies across all outcomes of interest, 19 reported the primary efficacy outcome of mosquito mortality (Table 2).

### 3.2. Included Studies

Table 2 reports the characteristics of included studies evaluating the efficacy of disinsection according to study design and intervention with the primary reported outcome of mosquito mortality.

### 3.3. Efficacy of Disinsection

Nineteen studies of disinsection of conveyances fulfilled study inclusion (Table 2), eighteen of which addressed aircraft disinsection only and one addressed both aircraft and transport truck disinsection [27]. No studies of mosquito disinsection efficacy on marine vessels, rail, or spacecraft were identified. Of nineteen studies of aircraft disinsection, five studies reported on pre-embarkation methods including residual disinsection (four studies) [28,29,30,31] and pre-boarding aerosol disinsection (two studies) [28,29], while eleven reported on the methods of “blocks-away” [27,32,33,34,35,36,37,38,39,40,41] and two on “top-of-descent” spraying [28,42]. An additional four studies reported on alternate methods of disinsection, including immediately after take-off [43] or upon arrival [42], or other unparticularized methods [44,45]. Nine of the nineteen studies included a comparator arm of unexposed mosquitoes [27,28,32,34,35,37,39,40,41], and as such permitted calculation of the main efficacy outcomes of both absolute and relative mosquito mortality, with calculated odds ratios and relative risks of mortality in the intervention versus control groups.

Of the nine comparator studies included, four evaluated one of the insecticides currently recommended for use in aircraft disinsection procedures as follows: four studies evaluated 2% d-phenothrin [27,28,35,41] and no studies evaluated 2% 1R-trans-phenothrin or 2% permethrin. Additionally, three studies evaluated 0.10–2% resmethrin [32,39,40], one study evaluated 1–2% allethrin [39], two studies evaluated 0.05–2% bioresmethrin [32,39], and four studies evaluated various concentrations of pyrethrins in combination with Tropital synergist (1.60–2.70%) [32,39] or DDT (1.17–3%) [32,34,37].

Of the nine comparator studies included, seven evaluated the efficacy of aircraft disinsection against *Aedes* spp. of mosquitoes [27,28,32,35,37,39,41], six against *Anopheles* spp. of mosquitoes [27,28,35,37,39,41], and six against *Culex* spp. of mosquitoes [27,28,34,35,37,40]. Amongst comparator trials evaluating aircraft disinsection efficacy, five studies used Boeing aircraft 707, 727, and 747 [27,28,34,35,39], one used Airbus 310 aircraft [28], three used DeHavilland D-6, D-8, or Comet aircraft [32,37,39], two used Lockheed aircraft [40,41], and one study used each of BAC aircraft [39], Vickers Viscount aircraft [37], and Sud Caravelle aircraft [37]. Amongst comparator (i.e., mosquito controlled) trials evaluating disinsection efficacy, eight studied the “blocks-away” methodology [27,32,34,35,37,39,40,41] and one studied pre-embarkation aerosol spraying [28] as well as pre-embarkation residual application to surfaces [28].

### 3.4. Summary of Methodological Quality and Risk of Bias Assessment

**Figure 3:**
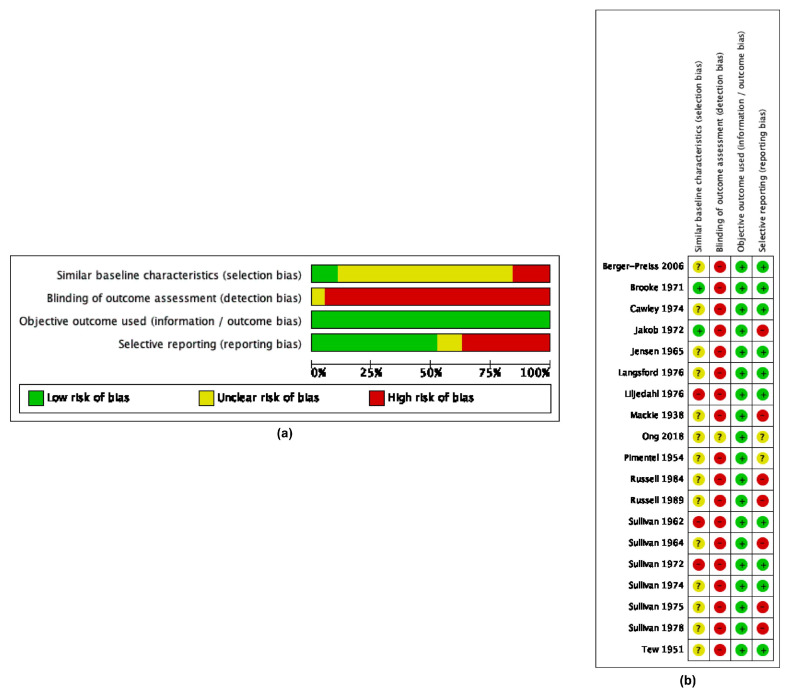
Risk of bias assessments for studies reporting efficacy of vehicular mosquito disinsection by (**a**) bias item and (**b**) study. References cited: [27,28,29,30,31,32,33,34,35,36,37,38,39,40,41,42,43,44,45].

Collectively, the studies of aircraft disinsection reporting mosquito mortality were of generally low quality with high risk of bias (Figure 3, Summary of Findings Table 3). Additionally, the composite methodological quality score as ascertained by adherence to the WHO’s published guidelines [26] for studies evaluating the efficacy of aircraft disinsection was 33.30%, ranging from 18.20% to 60.50% (Table 2 and Table 3).

**Table 2:** (**a**) Characteristics of included studies examining the efficacy of disinsection of vehicular conveyances. (**b**) Additional study characteristics of included studies examining the efficacy of disinsection of vehicular conveyances.

(**a**)								
**Author (Year)**	**Study** **Design**	**Country Setting**	**Conveyance**	Species *	**Sample Size °**	**Insecticide Used**	**Formulation**	**Disinsection Method**
Berger-Preiss (2006) [28]	Experimental Trial with comparator arm ‡	Germany	Grounded passenger aircraft (Airbus A310 and Boeing 747-400)	*Ae. aegypti*, *Anopheles stephensi*, *Culex pipiens*	Total: 9566; Exposed: 8921; Unexposed: 645; Initial: 1849; Residual: 7717; *A.a.* 4219; *A.s.* 1177; *C.p.* 4170	d-phenothrin	2% d-phenothrin	Simulated pre-flight and top-of-descent spraying
Brooke (1971) [32]	Experimental Trial with comparator arm ‡	UK	Grounded passenger aircraft (De Havilland Comet 4C)	*Ae. aegypti*	Total: 8000; Exposed: 7200; Unexposed: 800	Bioresmethrin, resmethrin, pyrethrins, DDT, bioallethrin, Tropital	Bioresmethrin: 0.05%, 0.075%, 0.10%, 0.25%; resmethrin: 0.10%, 0.25%, 0.50%; 0.40% pyrethrins + 3% DDT; 0.45% pyrethrins 0.45% + 2.7% Tropital	Simulated blocks-away with no passenger present
Cawley (1974) [33]	Experimental Trial	US	Commercial passenger aircraft (Boeing 707, Boeing 727)	*Culex pipiens fatigans*	ND	Bioresmethrin, resmethrin, S-2539 Forte	Bioresmethrin: 2% with 5% ethanol; resmethrin: 0.30%, 1.20%, and 2% with 5% ethanol; S-2539 Forte: 0.30%, 1.20%, and 2%	Blocks-away
Jakob (1972) [29]	Experimental Trial	US	Empty trailer trucks and unoccupied propeller-driven passenger aircrafts	*Ae. aegypti*, *Anopheles albimanus*, *Anopheles quadrimaculatus*	ND	Bromophos, carbaryl, chlorpyrifos, DDT, d-trans allethrin, fenitrothion, fenthion, gardona, mobam, Propoxur, pyrethrins, resmethrin, G-1707, G-1729, G-1730, G-1731	Micronized Dusts: 46.40% bromophos; 10% and 40% chlorpyrifos; 20% chlorpyrifos + 12.80% resmethrin; 13.30% chlorpyrifos + 8.50% resmethrin + 21.30% propoxur; 10% chlorpyrifos + 6.40% resmethrin + 16% propoxur + 20% gardona; 42.50% DDT + 42.50% carbaryl; 14% d-trans allethrin; 26.10% fenitrothion; 20.20% fenthion; 80% gardona; 83.30% mobam; 64% propoxur; 2.80% pyrethrins; 17% and 25.50% resmethrin; Aerosols: G-1707 (2.25% pyrethrins + 2.70% Tropital); G-1729 (2.25% pyrethrins + 2.70% sulfoxide); G-1730 (11% d-trans allethrin); G-1731; 7.50% resmethrin	Simulated trials of residual and pre-flight spraying, without passengers on board
Jensen (1965) [44]	Experimental Trial	US	Commercial passenger aircraft (DC-6B)	*An. quadrimaculatus*	ND	Dichlorvos vapour	Air concentration ranged from 0.13–0.25 ug/L dichlorvos	Disinsection anytime while aircraft is closed, and ventilation system is on
Langsford 1976 [34]	Experimental Trial with comparator arm ‡	Australia	Passenger aircraft (Boeing 747)	*Cx. fatigans*	Total: 330; Exposed: 260; Unexposed: 70	Pyrethrins	0.40% pyrethrins + 1.60% piperonyl butoxide, with 10% iso-paraffin solvents and Freon 11 + 12 as propellants	Blocks-away followed by saturation after disembarking
Liljedahl (1976) [35]	Experimental Trial with comparator arm ‡	US	Commercial passenger aircraft (Boeing 707, Boeing 727)	*Ae. aegypti*, *Aedes taeniorhynchus*, *An. quadrimaculatus*, *An. stephensi*, *Cx. pipiens fatigans*	Total: 5773; Exposed: 4677; Unexposed: 1096; *A.a.* 662; *A.t.* 2483; *A.q.* 1757; *A.s.* 351; *C.p.f.* 520	d-phenothrin	2% (+)-phenothrin in a 3:17 ratio of Freon-11 to 12 (break-off tip cans) and 2% (+)-phenothrin in a 1:1 ratio of Freon-11 to 12 (340 g cans with vertical release valves)	Blocks-away
Mackie (1938) [43]	Experimental Trial	UK	Passenger aircraft (Imperial flying boat)	ND	ND	Deskito (pyrethrum)	Pyrethrum water-based (1:14) insecticide with paraffin	Immediately after take-off
Ong (2018) [30]	Experimental Trial	Australia	Simulated aircraft environment	*Ae. aegypti* with 996P/1023G kdr mutation	ND	Permethrin	0.20 g/m^2^ as target dose of permethrin	Residual treatment
Pimentel (1954) [31]	Experimental Trial	US	Commercial aircraft (Convair-240, DC-3)	*Ae. aegypti*	~200	DDT, lindane	Formulation not specified; insecticides were dissolved in methylcyclohexane	Residual treatment
Russell (1984) [36]	Experimental Trials ^	Australia	Passenger aircraft (Boeing 707, Boeing 747)	*Culex quinquefasciatus*	1975–1976: ND; 1978: ND; 1980: 1500	d-phenothrin, pyrethrins	1975–1976: 0.40% pyrethrins + 1.60% piperonyl butoxide; 1978: 2% d-phenothrin 2%; 0.40% pyrethrins + 1.60% piperonyl butoxide; 0.40% pyrethrins + 1.60% piperonyl butoxide + 0.40% d-phenothrin; 1980: 2% d-phenothrin	Blocks-away
Russell (1989) [42]	Experimental Trials #	Australia	Passenger aircraft (Boeing 747, Boeing 767)	*Cx. quinquefasciatus*	20 per test site with 10–12 test sites per flight	d-phenothrin	2% d-phenothrin	Top-of-descent and on-arrival spraying
Sullivan (1962) [37]	Experimental Trial with comparator arm ‡	Italy, Switzerland, UK, US	Passenger aircraft (Boeing 707, Caravelle, Comet 4B, DC-6, DC-8, Viscount)	*Ae. aegypti*, *Anopheles gambiae*, *An. stephensi*, *Cx. fatigans*	Total: 7855; Exposed: 6574; Unexposed: 1281; *A.a.* 3157; *A.g.* 243; *A.s.*: 1065; *C.f.* 3390	Pyrethrum extract(s), pyrethrins, DDT	SRA: 1.60% pyrethrum extract (25% pyrethrins), 3% DDT, 7.50% xylene, 2.90% odourless petroleum distillate, 42.50% Freon-12, 42.50% Freon-11; G-1480: 3.40% pyrethrum extract (20% pyrethrins), 1.17% DDT, 4.50% aromatic petroleum derivative solvents, 63.62% Freon-12, 27.31% Freon-11	Blocks-away
Sullivan (1964) [38]	Experimental Trial	Fiji, New Zealand, Philippines	Passenger aircraft (DC-3, DC-7C, DC-8, Fokker, Viscount,)	*Ae. aegypti*, *Ae. albopictus*, *Cx. fatigans*	ND	DDT, G-1492, pyrethrum extract(s), SRA	SRA: 1.60% pyrethrum extract (25% pyrethrins), 3% DDT, 7.50% xylene, 2.90% odourless petroleum distillate, 42.50% Freon-12, 42.50% Freon-11; G-1492: 6% pyrethrum extract (20% pyrethrins), 2% DDT, 8% xylene, 58.80% Freon-12, 25.20% Freon-11	Blocks-away
Sullivan (1972) [39]	Experimental Trial with comparator arm ‡	US (WHO)	Commercial jet passenger aircraft (Boeing 747, Boeing 707, BAC 111, CD-8, DC-9)	*Ae. aegypti*, *Anopheles litoralis*, *An. stephensi*, *Culex molestus*, *Cx. pipiens fatigans*, *Culex pipiens pallens*	Total: 5076; Exposed: 4308; Unexposed: 768; *A.a.* 2035; *A.l.* 138; *A.s.* 207; *C.m.* 198; *C.p.f.* 2223; *C.p.p.* 275	Bioresmethrin, G-1707, resmethrin, pyrethrum extract(s), Tropital, (+)-trans-allethrin	Resmethrin: 1.12% and 2.25% aerosols; bioresmethrin: 1% and 2% aerosols; (+)-trans-allethrin: 1.11% and 2.22% aerosols; G-1707: 2.25% pyrethrum extract (20% pyrethrins, 2.70% Tropital synergist, 10.05% petroleum distillate, 59.90% Freon-12, 25.50% Freon-11	Blocks-away
Sullivan (1974) [27]	Experimental Trial with comparator arm ‡	US	Tractor trailers and commercial aircraft (Boeing 707, Boeing 727)	*Ae. aegypti*, *An. quadrimaculatus*	Total: 1162; Exposed: 602; Unexposed: 560; Tractors: 450; Airplanes: 712; *A.a.* 701; *A.q.* 461	d-phenothrin	1.20% d-phenothrin and 2% d-phenothrin (both in propellants Freon 11 + 12 50:50)	Blocks-away without passengers
Sullivan (1975) [40]	Experimental Trial with comparator arm ‡	US	Jet passenger aircraft (C-141, Lockheed)	*Cx. quinquefasciatus*	Total: 378; Exposed: 315; Unexposed: 63	d-trans-resmethrin, resmethrin	1.20% resmethrin and 98.66% propellants 11 + 12 (ratios 50:50 and 30:70); 1.20% d-trans resmethrin and 98.67% propellants 11 + 12 (50:50)	Blocks-away
Sullivan (1978) [41]	Experimental Trial with comparator arm ‡	US	Jet aircrafts for pilot training (Lockheed)	*Ae. taeniorhynchus*, *An. quadrimaculatus*	Total: 453; Exposed: 285; Unexposed: 168; *A.t.* 132; *A.q.* 321	d-phenothrin	Water-based: 2.03% (+)-phenothrin (98.5%), 0.87% Span 80, 0.03% Tween 60, 30% propellants (80% isobutane, 20% propane), 67.07% deionized water; Freon-based: 2.09% (+)-phenothrin (95.8%), 97.91% propellants (1:1 Freon 11 + 12)	Blocks-away
Tew (1951) [45]	Experimental Trial	UK	Grounded Argonaut and Tudor type 2 Aircraft	*Ae. aegypti*	200	DDT, pyrethrins	CMR 1: 0.40% pyrethrins and 3% DD; CMR 2: 1.20% pyrethrins and 2% DDT; CMR 3: 0.40% pyrethrins + 2% DDT + 3% piperonyl butoxide; CMR 4: 0.40% pyrethrins and 3% piperonyl butoxide; Am MS: 1.20% pyrethrins and 2% DDT; Am. IS: 1.20% pyrethrins and 2% DDT	Simulated spraying in grounded aircraft, not specified
(**b**)								
**Author (Year)**	**Insecticide Resistance**		**Mosquito Mortality**				Adherence to WHO Disinsection Guidelines * *	
Berger-Preiss (2006) [28]			Twenty minutes after spraying (pre-embarkation method), mortality was 94–99.50% for *Ae. aegypti* and 100% for *An. Stephensi*. Residual efficacy of disinsection, assessed between 7–48 h after spraying, yielded mosquito mortality between 89–100% on horizontal surfaces and 13–100% on vertical surfaces. Mortality was 0–6% in control mosquitoes.				4/22 (18.18%)	
Brooke (1971) [32]			Disinsection with any insecticide yielded a mean mosquito mortality of 97–100%, compared with 12% in control studies. Mean mosquito mortality by insecticide was as follows: 97% for 0.05% bioresmethrin, 98% for 0.075% bioresmethrin, 99% for 0.10% bioresmethrin, 100% for 0.25% bioresmethrin, 99% for 0.10% resmethrin, 100% for 0.25% resmethrin, 100% for 0.50% resmethrin, 100% for 0.40% pyrethrins + 3% DDT, and 100% for 0.45% pyrethrins + 2.70% Tropital.				6/16 (37.50%)	
Cawley (1974) [33]			*Cx. pipiens* mortality was tested on seats, floors, and rack positions. Mean mosquito mortality across positions was as follows: 0.30% resmethrin (99.23, 65.42, 29.33), 0.30% S-2539 Forte (86.17, 61.92, 23.95), 1.20% resmethrin (100, 100, 0), 1.20% S-2539 Forte (91.73, 72.47, 71.36), 2% resmethrin (100, 97.69, 96.35), 2% S-2539 Forte (100, 100, 96.81), and 2% bioresmethrin (100, 100, 100).				5/16 (31.25%)	
Jakob (1972) [29]			All aerosol formulations achieved 100% mosquito mortality in both truck trailers and aircrafts. Direct application of micronized dusts 40% chlorpyrifos, 17% and 25.50% resmethrin, and 20% chlorpyrifos + 12.80% resmethrin achieved 100% mosquito mortality in both truck trailers and aircrafts. Tests of 64% propoxur and 2.80% pyrethrins achieved 100% mosquito mortality in truck trailers and 100% mortality in the front and center positions on aircrafts; however, mortality was decreased in rear positions (propoxur achieved 0–95% mortality in rear positions and pyrethrins achieved 46–49% in rear positions). Direct application of micronized dusts 83.30% mobam, 10% chlorpyrifos, 46.40% bromophos, 26.10% fenitrothion, 20.20% fenthion, 14% d-trans allethrin, 42.50% DDT + 42.50% carbaryl, 10% chlorpyrifos + 6.40% resmethrin + 16% propoxur + 20% gardona, and 13.30% chlorpyrifos + 8.50% resmethrin + 21.30% propoxur achieved 100% mosquito mortality in truck trailers. Mobam achieved 99% mortality and gardona achieved 88–100% mortality in truck trailers. Residual treatment with micronized dusts 40% chlorpyrifos, 25.50% resmethrin, 20% chlorpyrifos + 12.80% resmethrin, 10% chlorpyrifos + 6.40% resmethrin + 16% propoxur + 20% gardona, and 83.30% and mobam achieved 100% mosquito mortality in truck trailers. Tests of 64% propoxur achieved 100% mortality of *Anopheles* but only 80–96% mortality of *Aedes*, 17% resmethrin achieved 75–86% mortality of *Anopheles* and 0–70% mortality of *Aedes*, and 2.80% pyrethrins achieved 0–5% mortality in both species. Mortality was ‘negligible’ except three tests with 10–21% mortality of *Ae. albimanus*.				5.5/16 (34.38%)	
Jensen (1965) [44]			100% mortality of *An. quadrimaculatus* mosquitoes was achieved on all six 30 min flights in all tested compartments (pilot compartment, seat racks, galley, and baggage compartments). No mortalities occurred in control specimens.				2.5/10 (25%)	
Langsford 1976 [34]			Initial inflight spray at the end of the landing roll achieved 100% mortality in all stations at 12 and 24 h. Initial inflight spray followed by a second saturation spray after passengers disembarked also achieved 100% mortality in all stations at 12 and 24 h. Control mosquito mortality was 0% at 12 h and 5.71% at 24 h (four out of 70 control mosquitoes found dead). Authors suggest this was expected as mosquitoes had been in cups for 36 h by this point.				5/16 (31.25%)	
Liljedahl (1976) [35]			In a Boeing-727, application of 2% (+)-phenothrin from the break-off tip can achieved 100% mortality of *An. quadrimaculatus* and 98–100% mortality of *Ae. taeniorhynchus*. Application from the 340-g vertical-release can achieved 98–100% mortality of *An. quadrimaculatus* and 93–100% mortality of *Ae. taeniorhynchus*. Mortality of control mosquitoes was 12–13% of *An. quadrimaculatus* and 0–6% of *Ae. taeniorhynchus*. In a Boeing-707, application from the 340-g vertical-release can achieved 89–100% mortality of *An. stephensi*, 93–100% mortality of *Ae. aegypti*, and 82–100% mortality of *Cx. pipiens fatigans*. Mortality of control mosquitoes was 0% of *An. stephensi*, 1% of *Ae. aegypti*, and 0% of *Cx. pipiens fatigans*.				6/17 (35.29%)	
Mackie (1938) [43]			In one experiment, 100% mosquito mortality was achieved within 2–11 min of spraying. In a second experiment, all but two mosquitoes were dead by 24 h; the two mosquitos alive at 24 h died “an hour or so later.”				4.5/16 (28.13%)	
Ong (2018) [30]	Resistant *Aedes aegypti* colony at 100% 996P/1023G *kdr* mutation frequencies was used as a proxy for mosquitoes intercepted at Australian airports. No mortality data reported for resistant mosquito colony.		Bioassays performed on permeable surfaces with 0.20 g/m^2^ permethrin achieved mortality of <50% in susceptible mosquitoes exposed for 30 min. Patchily treated environments typical of treated aircraft cabins and holds do not result in the universal exposure of mosquitoes in simulated environments.				N/A °°	
Pimentel (1954) [31]			DDT applied to baggage compartments did not provide satisfactory killing of mosquitoes (75% mortality one week after treatment, down to 18% mortality five weeks after treatment). Lindane, at various concentrations, applied to baggage and passenger compartments achieved 98–100% mortality up to five weeks after treatment and 83–100% mortality up to eight weeks after treatment.				2.5/9 (27.77%)	
Russell (1984) [36]			1975–1976 trials (0.40% pyrethrins + 1.60% piperonyl butoxide): B707 trial (November 1975, Auckland/Sydney) achieved 100% mosquito mortality. B747 trials (November 1975, Auckland/Sydney, and March 1976, Melbourne/Sydney) achieved less than 100% mortality mosquito (actual percentage not specified); 1978 trials (2% d-phenothrin, 0.40% pyrethrins + 1.60% piperonyl butoxide, and 0.40% pyrethrins + 1.60% piperonyl butoxide + 0.40% d-phenothrin): in a parked B747 in the Sydney airport, 100% mortality was observed after 18 h in all but “one exception.” Authors report “virtually 100% mosquito mortality.” No stratification by insecticide formulation reported; 1980 trials (2% d-phenothrin): in B747 Standard and Combi aircrafts, 99.80% (1497/1500) mosquito mortality was achieved at 24 h at fixed stations. Eleven single “wild cups,” or randomly placed cups aiming to target less accessible locations in the aircraft, achieved 100% mortality (N not specified). Control mosquitoes had 0% mortality in 38 stations and 10–30% mortality in 8 stations (N not specified).				6/17 (35.29%)	
Russell (1989) [42]			February 1986 trials: B747-300 from Singapore to Sydney, disinsected with 2% d-phenothrin via top-of-descent spraying with the air conditioning on, achieved 100% mortality of *Culex* mosquitoes; 1986 trials: B747-200 from Singapore to an unspecified Australia airport, disinsected with 2% d-phenothrin via on-arrival spraying with the air conditioning on, achieved 100% mortality of *Culex* mosquitoes; July 1987 trials: B767 from Sydney to Brisbane, disinsected with 2% d-phenothrin via top-of-descent spraying, achieved 100% mortality of mosquitoes.				11.5/19 (60.50%)	
Sullivan (1962) [37]	On London flights, DDT-resistant and susceptible *Ae. aegypti* were used. SRA achieved 0–33% mortality in DDT-resistant strains (compared to 81–100% mortality in susceptible strains). G-1480 achieved 0% mortality in one cage and 100% mortality in two cages of DDT-resistant mosquitoes (compared to 0% mortality in one cage and 100% mortality in four cages of susceptible strains). In the cage with 0% mortality of both resistant and susceptible mosquitoes, it was placed directly in front of an air inlet; On Rome flights, DDT-resistant *Cx. fatigans* were used. SRA achieved 0–100% mortality in one flight, 58–100% mortality in another flight, and 33–87% mortality in a training flight. G-1480 achieved 100% mortality in one flight and 27–65% mortality in a training flight (only 3/5 intended dosage used on the training flight).		G-1480 achieved 100% mortality of susceptible and resistant mosquitoes in all but two trials (in one trial, only 3/5 of proper dosage was used; in another trial, the mosquito cages with decreased mortality were placed directly in front of an air inlet). Control mosquito mortality was between 0–5%; SRA achieved 90–100% mortality in most trials of susceptible mosquitoes. SRA failed to achieve adequate mortality of DDT-resistant *Aedes* or *Culex* mosquitoes (see Insecticide Resistance column). Control mosquito mortality was between 0–4%.				8.5/18 (47.22%)	
Sullivan (1964) [38]	In the Philippines trials (Philippine mosquitos), *Aedes* had an increased tolerance to DDT (2–3 times the level of normal strains), as determined by susceptibility testing. *Cx. fatigans* were presumed resistant to DDT. G-1492 was more effective than SRA against resistant Philippine mosquitoes, achieving 100% mortality in 4/5 flights versus 100% mortality in 9/14 flights using SRA.		G-1492 was more effective than SRA against resistant Philippine *Aedes* and *Culex* mosquitoes. G-1492 achieved 100% mortality in 4/5 flights (84–94% in remaining one flight). SRA achieved 100% mortality in 9/14 flights, 96–100% mortality in 2/9 flights, and 40–100% mortality in 2/9 flights; G-1492 and SRA both achieved 100% mortality in susceptible Fiji *Culex* mosquitoes. Controls “in general” had 0–25% mortality, but was reported as high as 36–57% in “very few tests.”				6.5/17 (38.24%)	
Sullivan (1972) [39]	*Cx. pipiens fatigans* resistant to DDT: 2% d-trans-allethrin achieved 100% mortality in the cabin and lavatory, and 0% mortality in the cockpit. G-1707 achieved 81% mortality in the cabin; *Cx. pipiens fatigans* resistant to organophosphates: 2% resmethrin achieved 99% mortality in the cabin.		Authors arbitrarily selected morality of 97% as an acceptable level for the cabin. Only three of average mortality levels were lower than 97% (1% resmethrin at 94.20%, 1% d-trans-allethrin at 96.40%, and G-1707 at 94.90%) and in each case the confidence interval contained the 97% point; Mortality in lavatories was acceptable with 1% resmethrin and 2% d-trans-allethrin (100%). Mortality in lavatories was not acceptable with 2% resmethrin (43.8%), 1% d-trans-allethrin (85.2%), 1% bioresmethrin (30%), or G-1707 (75%). There was no acceptable mortality in the cockpit when tested (2% resmethrin achieved 33.30% mortality and 2% d-trans-allethrin and 2% bioresmethrin both achieved 0% mortality). Control mosquito mortality was 0% in all trials except for three, with mortality ranging from 4–8%. In authors’ opinion, 2% resmethrin aerosol at blocks-away appears to be the optimal procedure for disinsecting aircraft.				6.5/18 (36.11%)	
Sullivan (1974) [27]			100% mortality of *An. quadrimaculatus* was achieved in aircraft and tractor trailers with 1.20% phenothrin; 0% mortality in controls. 100% mortality of *Ae. aegypti* was also achieved in tractor trailers with 1.20% phenothrin; however, 79% mortality was seen in controls and invalidated the results for *Ae. aegypti*; 100% mortality of *Ae. aegypti* and *An. quadrimaculatus* was achieved in Boeing aircraft (707 and 727) with 2% phenothrin. Control mosquito mortality was 0% for *Ae. aegypti* and 8% for *An. quadrimaculatus*.				5.5/19 (28.94%)	
Sullivan (1975) [40]			100% mortality of *Culex* mosquitoes was achieved on all three flights with use of 1.20% resmethrin and 1.20% d-trans-resmethrin. Control mosquito mortality ranged from 0–25% (0% on two flights, 8% on one flight, and 25% on one flight).				7.5/18 (41.66%)	
Sullivan (1978) [41]			100% mortality of *Ae. taeniorhynchus* and *An. quadrimaculatus* was achieved in all five trials with mosquitoes (two water-based aerosols at blocks-away, two freon-based aerosols at blocks-away, and one freon-based aerosol on a grounded aircraft). Control mosquito mortality was 6% in *Ae. taeniorhynchus* and 14% in *An. quadrimaculatus*.				5/18 (27.77%)	
Tew (1951) [45]			In the Heathrow experiments with caged mosquitoes, Am. MS achieved 71–85% mortality, CMR 1 achieved 83–100% mortality, CMR 2 achieved 85–100% mortality, and Am. IS achieved 85–100% mortality. Control mosquito mortality was observed at 36% in experiments 1–6 and 40% in experiments 7–10. In the Farnborough experiments, both caged and free-flying mosquitoes were used. On the first day of experiments with caged mosquitoes, CMR 1 (dose reduced from 15 g/1000 ft^3^ to either 5 or 10 g/1000 ft^3^) achieved 99.50–100% mortality and CMR 3 achieved 100% mortality. On the second day of experiments with caged mosquitoes, CMR 1 (dose reduced to 5 g/1000 ft^3^) achieved 46% mortality with a closure time of 3 min and 69% mortality with a closure time of 5 min. CMR 4 achieved 65% mortality (closure time 5 min). On both days with free-flying mosquitoes, CMR 1 achieved 100% mortality (four tests), CMR 3 achieved 100% mortality, and CMR 4 achieved 99.50% mortality. Caged control mosquito mortality was observed at 0–2% in mosquitoes exposed in aircraft and 0–7% in mosquitoes in untreated aircraft. Higher mortality was noted with 5-min closure versus 3-min closure. Higher mortality was observed in free-flying mosquitoes versus caged mosquitoes.				5.5/16 (34.38%)	

* For abbreviated genera, *Ae.* = *Aedes*, *An.* = *Anopheles*, *Cx.* = *Culex.* Species names are listed as reported in the study regardless of present-day nomenclature (e.g., *Culex pipiens fatigans* now known as *Culex quinquefasciatus*); ***°*** In detailing sample size by mosquito species, *A.a.* = *Aedes aegypti*, *A.t.* = *Aedes taeniorhynchus*, *A.g.* = *Anopheles gambiae*, *A.l.* = *Anopheles litoralis*, *A.s.* = *Anopheles stephensi*, *A.q.* = *Anopheles quadrimaculatus*, *C.f.* = *Culex fatigans* (*now known as Cx. quinquefasciatus*), *C.m.* = *Culex molestus*, *C.p.* = *Culex pipiens*, *C.p.f.* = *Culex pipiens fatigans* (*now known as Cx. quinquefasciatus*), *C.p.p.* = *Culex pipiens pallens*; **‡** Denotes experimental trials that have a comparator arm of unexposed mosquitos, with both numerator and denominator data available for mosquito mortality; ^ Russell (1984) [36] reports on the outcomes of three separate sets of trials: (i) 1975–1976, (ii) 1978, (iii) 1980. Primary data for each set of trials unavailable; # Russell (1989) [42] reports on the outcomes of three separate sets of trials (i) Feb 1986, (ii) 1986, (iii) July 1987. Primary data for each set of trials unavailable. **DDT:** dichlorodiphenyltrichloroethane; **ND**: no data; **SRA:** standard reference aerosol; **ug/L:** microgram per liter; ** Percent adherence to a formulated checklist representing WHO *Guidelines for testing the efficacy of insecticide products used in aircraft* (2012) [26]; ^ Russell (1984) [36] reports on the outcomes of three separate sets of trials: (i) 1975–1976, (ii) 1978, (iii) 1980. Primary data for each set of trials unavailable; # Russell (1989) [42] reports on the outcomes of three separate sets of trials (i) Feb 1986, (ii) 1986, (iii) July 1987. Primary data for each set of trials unavailable; °° Ong (2018) [30]: unable to calculate adherence to WHO disinsection guidelines as study methods could not be evaluated on abstract alone (no published full text available).

**Table 3:** Summary of findings—efficacy outcome.

**Insecticide Compared to Control (No Insecticide) During Disinsection of Conveyances**												
Population: mosquitoes												
Setting: aircrafts												
Intervention: disinsection												
Comparison: no disinsection												
Outcome: mosquito mortality												
Study Design: experimental trial with non-exposed (control) comparator group												
**Stratification**	**No. of Studies**	**Mortality Exposed (%)**	**Mortality Unexposed (%)**	**Relative Risk (95% CI)**	**Odds Ratio (95% CI)**	**Risk of Bias**	**Inc. ^a^**	**Ind. ^b^**	**Imp. ^c^**	**Certainty of Evidence (GRADE) ^d^**	**Overall Adherence to WHO Guidelines on Disinsection ^f^**	**References**
***Overall***												
All insecticides	9	28,819/31,371 (91.90%)	421/ 6528 (6.50%)	14.24 (12.99–15.63)	163.60 (147–182)	Very serious	High risk	High risk	Low risk	Very Low ⨁◯◯◯	33.30% (54/162)	Sullivan (1974) [27]; Berger-Preiss (2006) [28]; Brooke (1971) [32]; Langsford (1976) [34]; Liljedhal (1976) [35]; Sullivan (1962) [37]; Sullivan (1972) [39]; Sullivan (1975) [40]; Sullivan (1978) [41]
***Genus of Mosquito***												
*Aedes*	6	17,649/18,437 (95.70%)	154/ 2804 (5.50%)	17.43 (14.96–20.33)	384 (321.60–458.40)	Serious	High risk	High risk	Low risk	Low ⨁⨁◯◯	33.20% (36.50/110)	Berger-Preiss (2006) [28]; Sullivan (1974) [27]; Brooke (1971) [32]; Liljedhal (1976) [35]; Sullivan (1962) [37]; Sullivan (1972) [39]
*Anopheles*	4	4461/4523 (98.60%)	69/ 1047 (6.60%)	14.97 (11.94–18.82)	1005 (709.20–1424)	Very serious	High risk	High risk	Low risk	Very Low ⨁◯◯◯	33.30% (25/75)	Berger-Preiss (2006) [28]; Liljedhal (1976) [35]; Sullivan (1962) [37]; Sullivan (1972) [39]
*Culex*	6	8004/9787 (81.80%)	20/ 1452 (1.40%)	59.37 (38.61–91.55)	313.60 (202.2–486.5)	Very serious	High risk	High risk	Low risk	Very Low ⨁◯◯◯	34.40% (37.50/109)	Berger-Preiss (2006) [28]; Langsford (1976) [34]; Liljedhal (1976) [35]; Sullivan (1962) [37]; Sullivan (1972) [39]; Sullivan (1975) [40]
Pooled: genus	9	30,114/32,747 (92%)	243/ 5303 (4.60%)	20.07 (17.76–22.70)	237.60 (207.70–271.90)	Very serious	High risk	High risk	Low risk	Very Low ⨁◯◯◯	33.30% (54/162)	Sullivan (1974) [27]; Berger-Preiss (2006) [28]; Brooke (1971) [32]; Langsford (1976) [34]; Liljedhal (1976) [35]; Sullivan (1962) [37]; Sullivan (1972) [39]; Sullivan (1975) [40]; Sullivan (1978) [41]
***Method of Disinsection***												
Pre-embarkation (aerosol)	1	1595/1612 (99%)	0/ 237(0%)	∞ (2–∞)	43,306 (2596–722,509)	Serious	N/A	High risk	High risk	Very Low ^e^ ⨁◯◯◯	18.20% (4/22)	Berger-Preiss (2006) [28]
Pre-embarkation (residual)	1	5781/7309 (79.10%)	8/ 408 (2%)	40.34 (20.70–79.35)	178.20 (90.14–352.40)	Serious	N/A	High risk	High risk	Very Low ^e^ ⨁◯◯◯	18.20% (4/22)	Berger-Preiss (2006) [28]
Blocks-away	8	21,667/22,674 (95.60%)	235/ 4451 (5.30%)	18.10 (15.99–20.50)	385.10 (332.90–445.40)	Very serious	High risk	High risk	High risk	Very Low ⨁◯◯◯	35.70% (50/140)	Sullivan (1974) [27]; Brooke (1971) [32]; Langsford (1976) [34]; Liljedhal (1976) [35]; Sullivan (1962) [37]; Sullivan (1972) [39]; Sullivan (1975) [40]; Sullivan (1978) [41]
Pooled: method of disinsection	9	29,043/31,595 (91.90%)	243/ 5096 (4.80%)	19.28 (17.06–21.80)	226.80 (198.20–259.60)	Very serious	High risk	High risk	High risk	Very Low ⨁◯◯◯	33.30% (54/162)	Sullivan (1974) [27]; Berger-Preiss (2006) [28]; Brooke (1971) [32]; Langsford (1976) [34]; Liljedhal (1976) [35]; Sullivan (1962) [37]; Sullivan (1972) [39]; Sullivan (1975) [40]; Sullivan (1978) [41]
***Insecticide***												
Allethrin	1	328/378 (86.80%)	2/ 103 (1.90%)	44.69 (12.74–162.50)	264.10 (72.75–958.70)	Very serious	N/A	High risk	High risk	Very Low ^e^ ⨁◯◯◯	36.10% (6.50/18)	Sullivan (1972) [39]
Bioresmethrin	2	4435/4570 (97.10%)	102/ 1057 (9.70%)	10.06 (8.38–12.11)	305.10 (233.90–398.10)	Serious	High risk	High risk	High risk	Very Low ^e^ ⨁◯◯◯	36.80% (12.50/34)	Brooke (1971) [32]; Sullivan (1972) [39]
DDT-containing	3	5639/6319 (89.20%)	112/ 1940 (5.80%)	15.46 (12.93–18.52)	134.70 (109.60–165.60)	Serious	Low risk	High risk	Low risk	Low ⨁⨁◯◯	39% (19.50/50)	Brooke (1971) [32]; Langsford (1976) [34]; Sullivan (1962) [37]
d-phenothrin	4	12,687/14,274 (88.90%)	96/ 2169 (4.40%)	20.08 (16.53–24.43)	171.70 (139.10–212)	Serious	High risk	High risk	Low risk	Low ⨁⨁◯◯	27% (20.50/76)	Sullivan (1974) [27]; Berger-Preiss (2006) [28]; Liljedhal (1976) [35]; Sullivan (1978) [41]
Pyrethrins	4	6820/7523 (90.70%)	114/ 2044 (5.60%)	16.25 (13.61–19.44)	163.50 (133.30–200.40)	Serious	High risk	High risk	Low risk	Low ⨁⨁◯◯	38.20% (26/68)	Brooke (1971) [32]; Langsford (1976) [34]; Sullivan (1962) [37]; Sullivan (1972) [39]
Resmethrin	3	4549/4626 (98.30%)	107/ 1155 (9.30%)	10.60 (8.68–12.73)	572.60 (424.30–772.60)	Very serious	Low risk	High risk	High risk	Very Low ⨁◯◯◯	38.50% (20/52)	Brooke (1971) [32]; Sullivan (1972) [39]; Sullivan (1975) [40]
Pooled: insecticide	9	28,819/31,371 (91.90%)	421/ 6528 (6.50%)	14.24 (12.99–15.63)	163.60 (147–182)	Very serious	High risk	High risk	Low risk	Very Low ⨁◯◯◯	33.30% (54/162)	Sullivan (1974) [27]; Berger-Preiss (2006) [28]; Brooke (1971) [32]; Langsford (1976) [34]; Liljedhal (1976) [35]; Sullivan (1962) [37]; Sullivan (1972) [39]; Sullivan (1975) [40]; Sullivan (1978) [41]
***Aircraft***												
Airbus	1	5281/6826 (77.40%)	4/ 565 (0.70%)	109.30 (42.83–280.7)	426.40 (168.30–1080)	Serious	N/A	High risk	High risk	Very Low ^e^ ⨁◯◯◯	18.20% (4/22)	Berger-Preiss (2006) [28]
BAC	1	566/601 (94.20%)	2/148 (1.40%)	69.69 (19.65–253.60)	935.10 (256.10–3415)	Very serious	N/A	High risk	High risk	Very Low ⨁◯◯◯	36.10% (6.50/18)	Sullivan (1972) [39]
Boeing	5	8728/8839 (98.70%)	76/ 1784 (4.30%)	23.18 (18.63–28.90)	1748 (1301–2350)	Serious	High risk	High risk	High risk	Low ⨁⨁◯◯	29.30% (27/92)	Sullivan (1974) [27]; Berger-Preiss (2006) [28]; Langsford (1976) [34]; Liljedhal (1976) [35]; Sullivan 1972 [39]
Caravelle	1	230/330 (69.70%)	0/72 (0%)	∞ (2–∞)	332.60 (20.40–5421)	Very serious	N/A	High risk	High risk	Very Low ⨁◯◯◯	47.20% (8.5/18)	Sullivan (1962) [37]
De Havilland	3	12,145/12,667 (95.90%)	107/ 1882 (5.70%)	16.86 (14.05–20.29)	383.90 (310.10–475.30)	Very serious	Low risk	High risk	Low risk	Very Low ⨁◯◯◯	40.40% (21/52)	Brooke (1971) [32]; Sullivan (1962) [37]; Sullivan (1972) [39]
Lockheed	2	539/539 (100%)	28/219 (12.80%)	7.82 (5.71–9.44)	7250 (440.50–119,330)	Very serious	Low risk	High risk	High Risk	Very Low ⨁◯◯◯	34.7% (12.50/36)	Sullivan (1975) [40]; Sullivan (1978) [41]
Viscount	1	1330/1569 (84.80%)	4/ 258 (1.60%)	54.67 (21.63–140.20)	314.20 (122.50–806.10)	Very serious	N/A	High risk	High risk	Very Low ⨁◯◯◯	47.20% (8.50/18)	Sullivan (1962) [37]
Pooled: aircraft	9	28,769/31,321 (91.90%)	220/ 4913 (4.50%)	20.48 (18.02–23.35)	240 (208.50–276.20)	Very serious	High risk	High risk	Low risk	Very Low ⨁◯◯◯	33.30% (54/162)	Sullivan (1974) [27]; Berger-Preiss (2006) [28]; Brooke (1971) [32]; Langsford (1976) [34]; Liljedhal (1976) [35]; Sullivan (1962) [37]; Sullivan (1972) [39]; Sullivan (1975) [40]; Sullivan (1978) [41]

CI = confidence intervals; GRADE Working Group grades of evidence; Inc: Inconsistency; Ind: Indirectness; Imp: Imprecision; N/A: not applicable. ^a^: Inconsistency assigned to studies due to variance in point estimates and no/minimal overlap in confidence intervals; ^b^: Indirectness assigned to all studies due to use of non-WHO approved insecticides, obsolete passenger aircraft, and overall non-adherence to WHO published guidelines on testing efficacy of disinsection. Only one study (Berger-Preiss 2006) [28] used a WHO-approved insecticide and currently operational passenger aircraft model, but was considered high risk of indirectness due to poor adherence to WHO guidelines on testing disinsection (18.18%); ^c^: Indirectness assigned due to wide 95% confidence intervals; ^d^: All studies were downgraded at least one level of evidence based on inconsistency, indirectness, and imprecision; ^e^: Pre-embarkation stratified subgroups (under Method of Disinsection) and Airbus stratified subgroup (under Aircraft) were downgraded two levels of evidence based on high risk of indirectness (combination of insecticide and method tested—d-phenothrin and pre-embarkation/residual treatments—are not WHO recommended, and poor adherence to WHO disinsection testing guidelines) and imprecision (wide 95% confidence intervals). Bioresmethrin and allethrin (under Insecticide) were also downgraded two levels of evidence given high risk in inconsistency for bioresmethrin (large variance between point estimates of studies), indirectness for both (not a WHO-approved insecticide, obsolete passenger aircraft, poor adherence to WHO disinsection testing guidelines), and imprecision for both (wide 95% confidence intervals); ^f^: Percent adherence to a formulated checklist representing WHO Guidelines for testing the efficacy of insecticide products used in aircraft (2012). The value listed is an aggregate average of all studies represented in each stratified line item.

### 3.5. Quantitative and Qualitative Synthesis: Summary of Findings—Efficacy

A quantitative synthesis of aircraft disinsection efficacy can be found in Summary of Findings Table 3. Collectively across comparator trials of aircraft disinsection efficacy, the odds of mosquito mortality in the experimental (exposed) arms compared to control (unexposed) arms (i.e., the odds ratio) was 163.60 (95% confidence interval [CI] 147–182) and the risk of mosquito death in the exposed versus unexposed arms (i.e., the relative risk) was 14.24 (95% CI 12.99–15.63) (Table 3).

**Figure 4:**
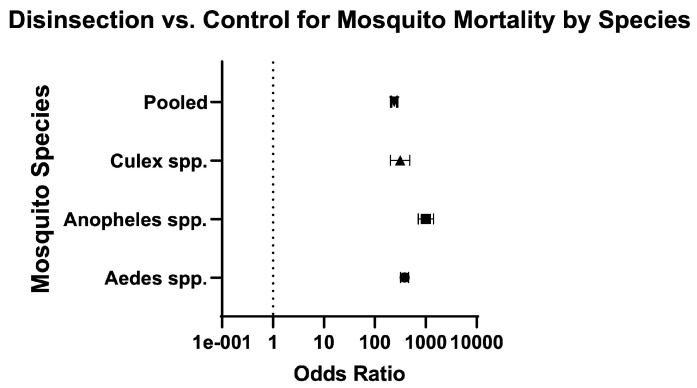
Forest plot of odds ratios of disinsection efficacy according to mosquito species. Included studies: [27,28,32,34,35,37,39,40].

**Figure 5:**
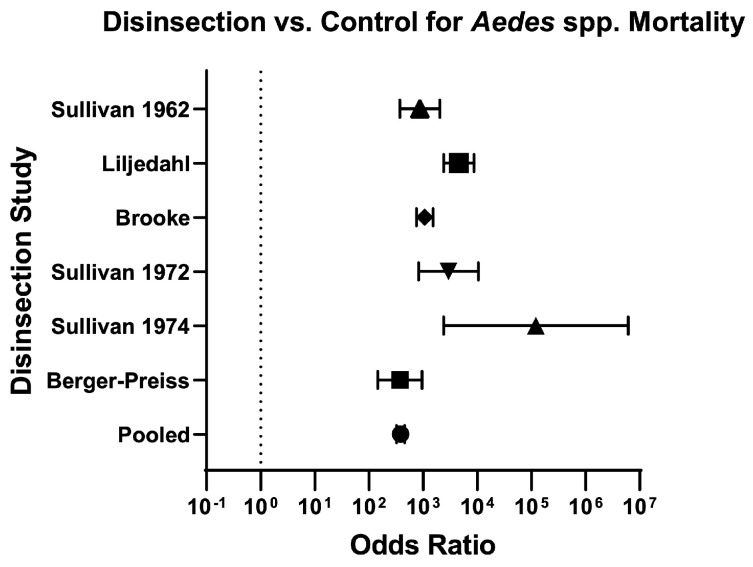
Forest plot of odds ratios of disinsection efficacy for *Aedes* spp. mosquitoes. Included studies: [27,28,32,35,37,39].

**Figure 6:**
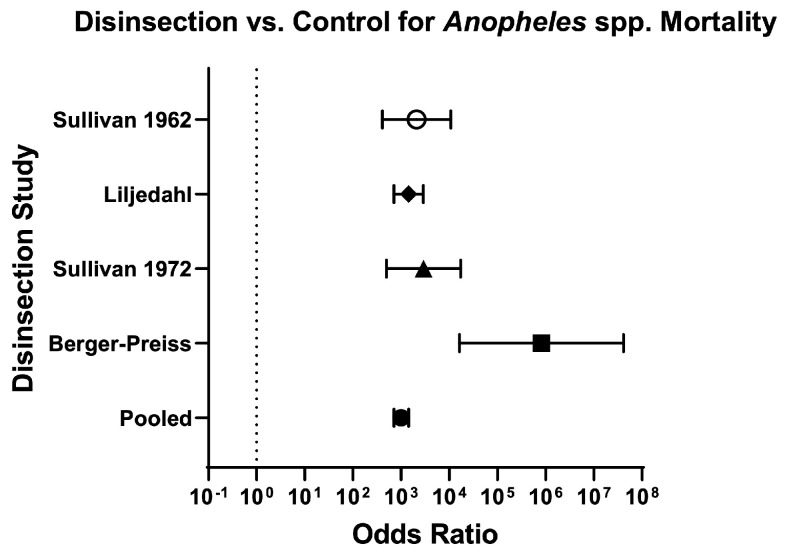
Forest plot of odds ratios of disinsection efficacy for *Anopheles* spp. mosquitoes. Included studies: [28,35,37,39].

**Figure 7:**
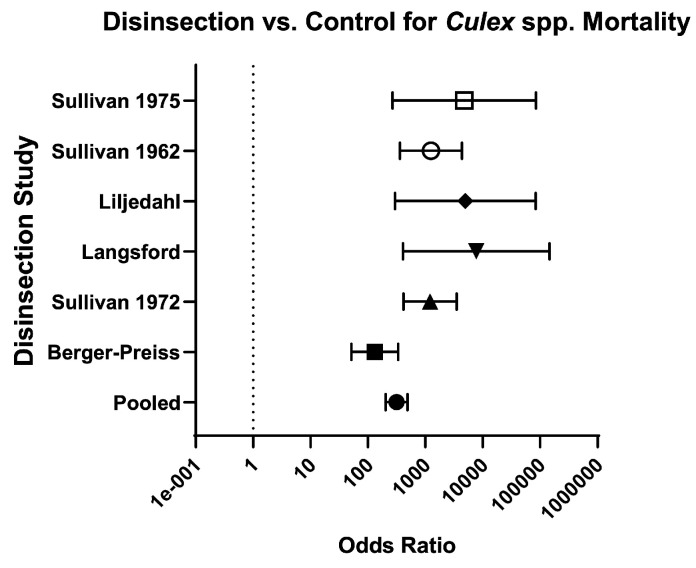
Forest plot of odds ratios of disinsection efficacy for *Culex* spp. mosquitoes. Included studies: [28,34,35,37,39,40].

Across mosquito genera, the odds of mosquito mortality for disinsection versus control was 237.60 (95% CI 207.70–271.90) and relative risk was 20.07 (95% CI 17.76–22.70) (Table 3; Figure 4). For *Aedes* species of mosquitoes, the odds of mosquito mortality for disinsection versus control was 384 (95% CI 321.60–458.40) with relative risk of 17.43 (95% CI 14.96–20.33) (Figure 5). For *Anopheles* species of mosquitoes, the odds of mosquito mortality for disinsection versus control was 1005 (95% CI 709.20–1424) with relative risk of 14.97 (95% CI 11.94–18.82) (Figure 6). For *Culex* species of mosquitoes, the odds of mosquito mortality for disinsection versus control was 313.60 (95% CI 202.20–486.50) with relative risk of 59.37 (95% CI 38.61–91.55) (Figure 7).

**Figure 8:**
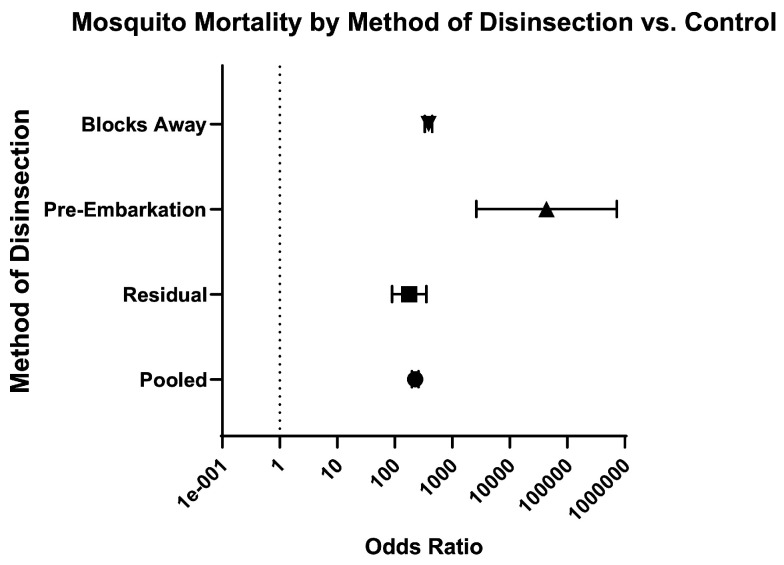
Forest plot of odds ratios of disinsection efficacy according to method of disinsection. Included studies: [27,28,32,34,35,37,39,40,41].

**Figure 9:**
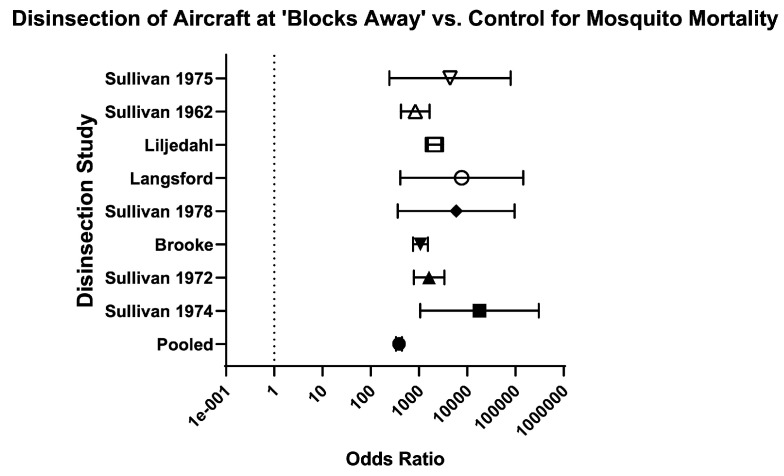
Forest plot of odds ratios of disinsection efficacy for “blocks-away” disinsection. Included studies: [27,32,34,35,37,39,40,41].

Across methods of disinsection, the odds of mosquito mortality for disinsection versus control was 226.80 (95% CI 198.20–259.60) and relative risk was 19.28 (95% CI 17.06–21.80) (Table 3; Figure 8). For pre-embarkation residual disinsection, the odds of mosquito mortality for disinsection versus control was 178.20 (95% CI 90.14–352.40) with relative risk of 40.34 (95% CI 20.70–79.35). For pre-embarkation aerosol spraying, the odds of mosquito mortality for disinsection versus control was 43,306 (95% CI 2,596–722,509) with relative risk of infinity (95% CI 2 to infinity). For “blocks-away” disinsection, the odds of mosquito mortality for disinsection versus control 385.10 (95% CI 332.90–445.40) with relative risk of 18.10 (95% CI 15.99–20.50) (Figure 9). No comparator trials (i.e., with an unexposed mosquito control arm) for “top-of-descent” spraying were identified.

**Figure 10:**
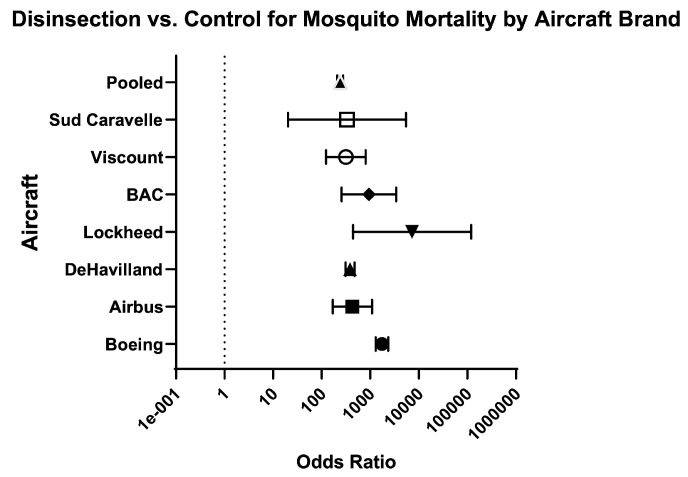
Forest plot of odds ratios of disinsection efficacy according to make of aircraft. Included studies: [27,28,32,34,35,37,39,40,41].

**Figure 11:**
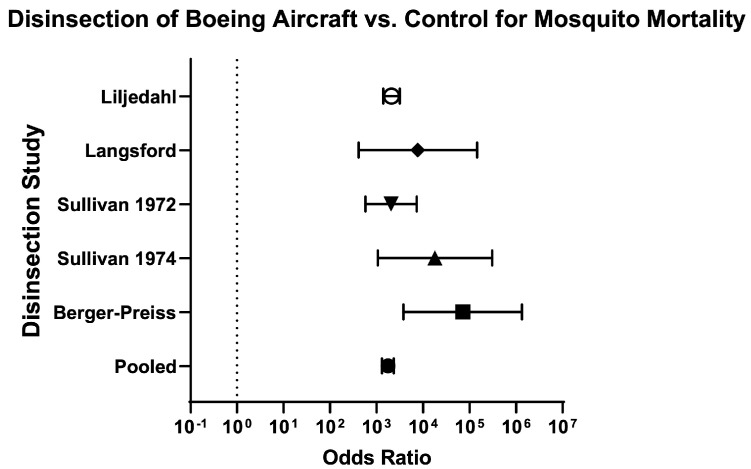
Forest plot of odds ratios of disinsection efficacy for Boeing aircraft. Included studies: [27,28,34,35,39].

**Figure 12:**
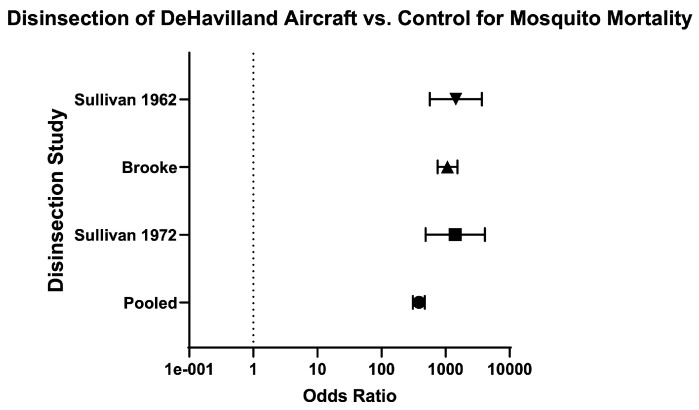
Forest plot of odds ratios of disinsection efficacy for De Havilland aircraft. Included studies: [32,37,39].

**Figure 13:**
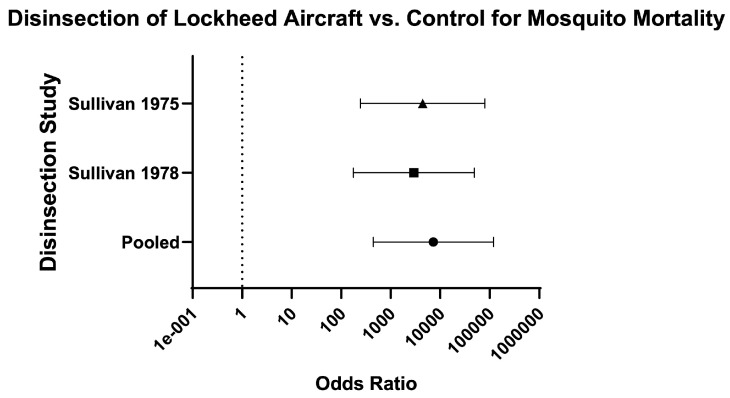
Forest plot of odds ratios of disinsection efficacy for Lockheed aircraft. Included studies: [40,41].

Across aircraft models, the odds of mosquito mortality for disinsection versus control was 240 (95% CI 208.50–276.20) and the relative risk was 20.48 (95% CI 18.02–23.31) (Table 3; Figure 10). For models of Boeing aircraft (707, 727, and 747), the odds of mosquito mortality for disinsection versus control was 1748 (95% CI 1301–2350) with relative risk of 23.18 (95% CI 18.63–28.90) (Figure 11). For models of Airbus aircraft (310), the odds of mosquito mortality for disinsection versus control was 426.40 (95% CI 168.30–1080) with relative risk of 109.30 (95% CI 42.83–280.70). For models of De Havilland aircraft (D-6, D-8, and Comet), the odds of mosquito mortality for disinsection versus control was 383.90 (95% CI 310.10–475.30) with relative risk of 16.86 (95% CI 14.05–20.29) (Figure 12). For models of Lockheed aircraft, the odds of mosquito mortality for disinsection versus control was 7250 (95% CI 440.50–119,330) with relative risk of 7.82 (95% CI 5.71–9.44) (Figure 13). Odds ratios and relative risks for mosquito mortality in BAC, Vickers Viscount, and Sud Caravelle models of aircraft are as noted in the Efficacy Summary of Findings table and all-aircraft Forest plot (Table 3; Figure 10).

**Figure 14:**
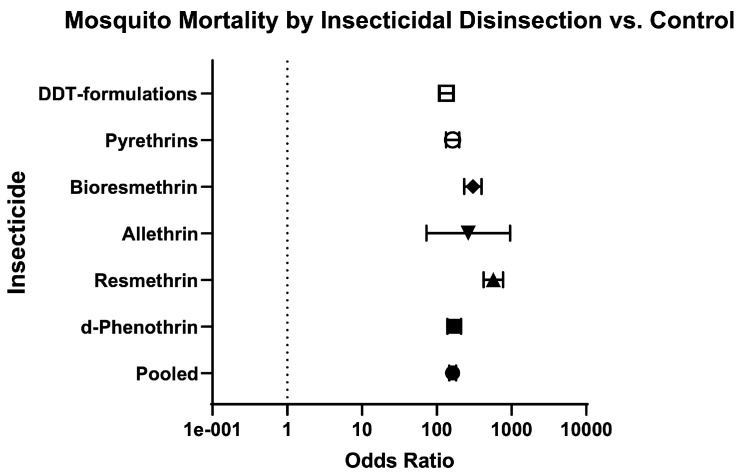
Forest plot of odds ratios of disinsection efficacy according to insecticide. Included studies: [27,28,32,34,35,37,39,40,41].

**Figure 15:**
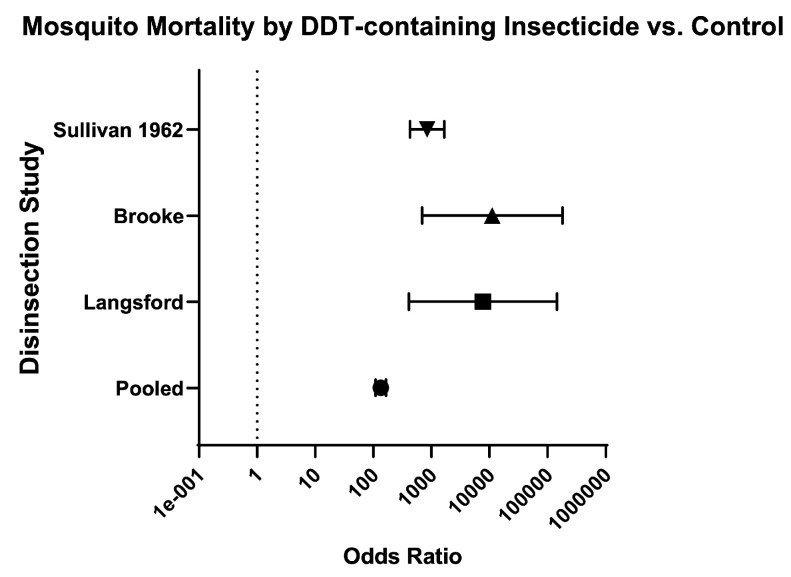
Forest plot of odds ratios of disinsection efficacy for DDT-containing insecticides. Included studies: [32,34,37].

**Figure 16:**
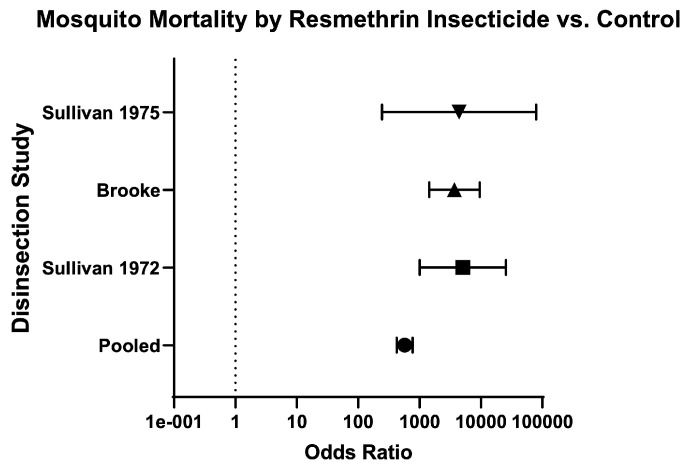
Forest plot of odds ratios of disinsection efficacy for resmethrin insecticide. Included studies: [32,39,40].

**Figure 17:**
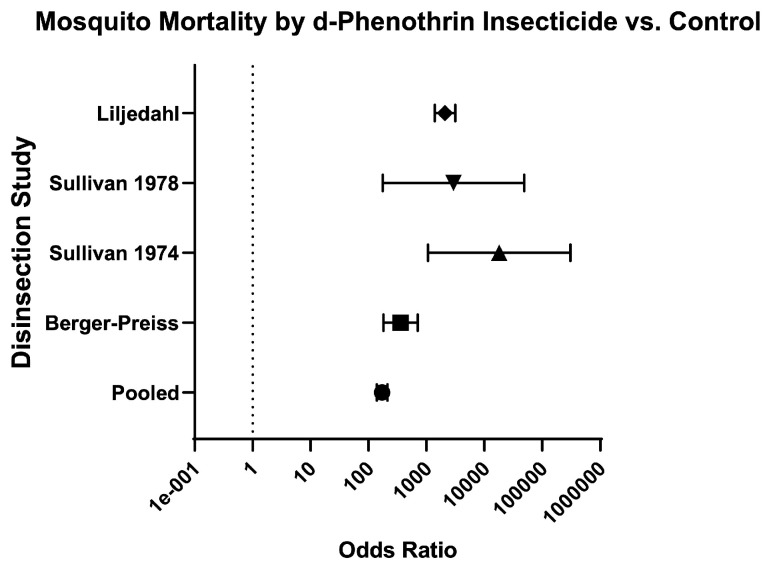
Forest plot of odds ratios of disinsection efficacy for d-phenothrin insecticide. Included studies: [27,28,35,41].

**Figure 18:**
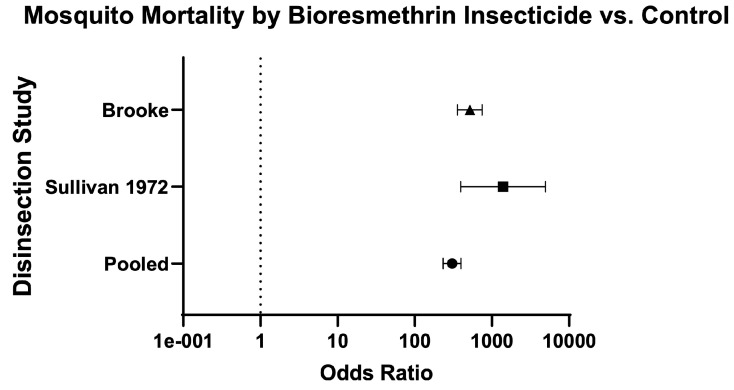
Forest plot of odds ratios of disinsection efficacy for bioresmethrin insecticide. Included studies: [32,39].

**Figure 19:**
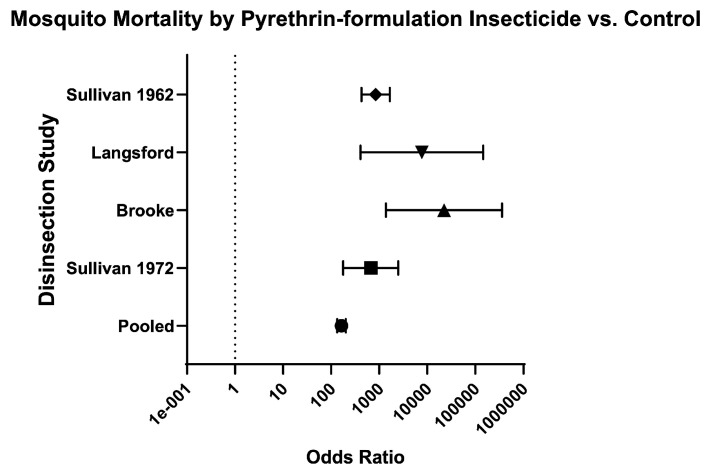
Forest plot of odds ratios of disinsection efficacy for pyrethrin- (pyrethrum-extract) containing insecticides. Included studies: [32,34,37,39].

Finally, the odds of mosquito mortality for disinsection versus control was variable across different insecticides (Table 3; Figure 14), ranging from 134.70 (95% CI 109.60–165.60) for DDT-containing formulations (Figure 15) to 572.60 (95% CI 424.30–772.60) for resmethrin formulations (Figure 16), with relative risks of 15.46 (95% CI 12.93–18.52) and 10.61 (95% CI 8.88–12.73), respectively (Table 3; Figure 14). The only WHO-recommended formulation to be tested in a mosquito-controlled comparator trial was 2% d-phenothrin, which yielded an odds of mosquito mortality for disinsection versus control of 171.70 (95% CI 139.10–212) across four studies [27,28,35,41] with relative risk of 20.08 (95% CI 16.53–24.43) (Figure 17). For the insecticide allethrin, the odds of mosquito mortality for disinsection versus control was 264.10 (95% CI 72.75–958.70) with relative risk of 44.69 (95% CI 12.74–162.50). For the insecticide bioresmethrin, the odds of mosquito mortality for disinsection versus control was 305.10 (95% CI 233.90–398.10) with relative risk of 10.06 (95% CI 8.38–12.11) (Figure 18). For pyrethrin-containing formulations (combined with either Tropital synergist or DDT), the odds of mosquito mortality for disinsection versus control was 163.50 (95% CI 133.30–200.40) across four studies [32,34,37,39] with relative risk of 16.25 (95% CI 13.61–19.44) (Figure 19).

## 4. Discussion

This systematic review identified only four mosquito-controlled comparator trials investigating the efficacy of a WHO-recommended insecticide formulation (2% d-phenothrin) for the purpose of aircraft disinsection, which supported a high degree of insecticidal efficacy. Studies on 2% permethrin or 2% 1R-trans-phenothrin were not identified. Furthermore, no studies (of 19 included) of any insecticide identified by the systematic review adhered to WHO recommendations for conduct of such experimental trials and as such the true efficacy of such disinsection procedures in a real-life context is uncertain. The evidence base upon which current disinsection guidance is predicated predates the publication of WHO’s process-oriented guidelines for investigating disinsection efficacy [26], and as such, the evidence base warrants updating to current methodological and reporting standards. Many knowledge gaps remain, particularly around the feasibility of performing disinsection procedures in accordance with current guidance (e.g., with air conditioner switched off while passengers are on board), environmental impacts, and generation of insecticide resistance amongst mosquitoes.

Developing, testing, and establishing standards and performance criteria for non-chemical aircraft cabin disinsection methods (e.g., UV-based, electrostatic, sonic, impregnated curtains, etc.) that minimize impacts of disinsection on human health would be a worthwhile research agenda. The current guidelines on methodological aspects to include in studies of disinsection efficacy are process oriented and we could locate no corollary guidance document for evaluating aircraft and conveyance disinsection safety and toxicity. Similarly, testing the feasibility, efficacy, and safety of already available electrostatic sprayers for residual disinsection, which reduce the amount of spray liquid to be applied in aircraft and other conveyances, could be considered. Future disinsection research endeavours should also consider advancements in aircraft size and design (for example, multi-deck aircraft), requiring that exposure assessments address characteristics such as different aircraft airflow systems and uncertainties in onboard ventilation performance against standards. Moreover, identifying the optimal methods and procedures for effective and safe marine vessel disinsection—taking into account variable vessel length, cabin composition, and duration of voyage—is a worthwhile endeavour, given the absolute dearth of such data.

Limitations of this systematic review include a highly complex literature base representing disinsection procedures across a variety of mosquito species, aircraft, procedures, and formulations. However, the directionality of effect was consistent and large across studies. The generally low level of adherence to recommended procedures for designing and implementing studies of disinsection efficacy limits the generalizability and confidence in summary estimates of effect. Further, the lack of consistently reported information regarding ambient cabin temperature, humidity, air pressure, and precise duration over which time insecticide canisters were discharged potentially introduces further bias in the reported results. The absence of comparator trials of marine vessel, rail, and motor car disinsection limits our ability to comment on the effectiveness of such procedures. Furthermore, the lack of literature extending—in a causal way—the effectiveness of disinsection to actual exportation events as well as development of novel breeding foci in destination countries means that the true contribution of aircraft disinsection to mitigation of mosquito dispersal events is unknown. Finally, the lack of controlled trials evaluating WHO-recommended disinsection formulations other than 2% d-phenothrin limits our ability to comment on the efficacy of currently and commonly used disinsection procedures.

In conclusion, the high efficacy of disinsection procedures evaluated in the studies reported herein must be interpreted in the context of a high degree of non-adherence to published guidelines on necessary methodological aspects of such trials. No included studies adhered fully to recommended study procedures and as such the generalizability, accuracy, and precision of the reported findings are uncertain. Future high-quality and guidelines-adherent trials are required to fully understand the efficacy of aircraft, marine vessel, and ground transport disinsection in preventing dispersal of mosquitoes via international conveyances.

## Data Availability

The original contributions presented in this study are included in the article/Supplementary Material. Further inquiries can be directed to the corresponding author.

## Supplementary Materials

The following supporting information can be downloaded at: https://www.mdpi.com/article/10.3390/insects16090911/s1, Table S1: Countries and territories with Aircraft Disinsection Regulations; Table S2: WHO-recommended aircraft cabin disinsection procedures; Table S3: Disinsection Regulations for Marine/Submarine Conveyances; Table S4: Efficacy of Aircraft Disinsection Checklist. References [46,47,48,49,50,51,52,53] are included in the Supplementary Material.
