# Supplementary material for: A Systematic Review of Aircraft Disinsection Efficacy
Source: Insects. 2025 Sep 1;16(9):911. doi: 10.3390/insects16090911 (PMC12470303; doi:10.3390/insects16090911)
Supplement: Supplementary file 1 [file insects-16-00911-s001.zip › insects-3810555-supplementary.pdf]

Table S1. Countries and territories with Aircraft Disinsection Regulations [1,2,3]

Country	Regulation
United States of America (Arrival) Guidelines for Disinsection 2021: https://www.transportation.gov/airconsumer/spray	
Ecuador*	Countries requiring the disinsection of all in-bound flights with an aerosolized spray while passengers are on board
Grenada	
Guyana	
India	
Kiribati	
Madagascar	
Panama	
Seychelles	
Unitedd Republic of Tanzania (the)	
Timor-Leste	
Trinidad and Tobago	
Uruguay	
Zimbabwe	
Australia	Countries requiring the disinsection of all in-bound flights but allowing the residual method of disinsection
Barbados	
Chile	
Cook Islands	
Fiji	
Jamaica	
New Zealand	
Czech Republic	Areas of contagious diseases
Egypt	Zika-affected countries
France	Areas of malaria, yellow fever and dengue fever
China, Hong Kong SAR	All incoming aircraft from Zika-affected countries designated as WHO Category 1 or Category 2
Indonesia	Areas affected by any sort of infectious or contagious disease**
Italy	All aircraft coming from areas affected by Zika virus transmission and areas where the *Aedes aegypti* carrier is present

Mauritius	Flights from African continent, Asia and sub regions, the Middle East and islands of the Indian Ocean, and any other country where mosquito borne diseases are prevalent
Macau	Flights from areas of major infectious disease or Zika-affected countries
Palau	Non-US carriers from Korea, Hong Kong, Macau and Thailand
Peru	Some in-country flights
Republic of Korea (the)	30 countries, not including the United States
South Africa	Areas of malaria or yellow fever
Switzerland	Intertropical Africa
Taiwan, China	Incoming flights from areas with arbovirus vectors *Aedes aegypti* and *Ae. albopictus*
Thailand	Areas of yellow fever
United Kingdom of Great Britain and Northern Ireland	Malarial countries and countries with confirmed transmission of Zika (Voluntary)
Canada (Arrival) Guidelines for Disinsection ~2017: https://www.acta.ca/news-releases/cb0206 https://www.aircanada.com/ca/en/aco/home/plan/peace-of-mind/travel-tips.html#/	
Australia	Pre-embarkation Method (Primary Method) – This method takes place without passengers or crew on board, and is performed or supervised by a Certificate holder.
New Zealand	On Arrival Method (Alternate Method) – This method takes place before passengers have disembarked and the doors have been opened. Crew walk through the cabins discharging approved single-shot aerosols in the prescribed dosage.
Aruba	Top of descent method: This method is similar to the "On arrival method", except that it is carried out at the top of the aircraft's descent, just before it starts preparations for landing.
Barbados	
Buenos Aires (Argentina)[3]	
Cuba	
Guadeloupe	
Jamaica	
Martinique	
Puerto Rico	
St-Lucia[1]	

St. Vincent and the Grenadines	
Santiago (Chile)[2]	
Trinidad and Tobago	
Turks & Caicos	

* only Galapagos and Interislands

**Indonesia is sensitive to infectious diseases due to population health and climate which helps propagate infectious disease such as SARS and influenza from China, MERS CoV from the Middle East, and HIV from areas in Africa. [4]

[1] Spraying only required if flight transits at another destination before arrival.

[2] Spraying is required YYZ - SCL before landing at Santiago, Chile, and SCL - EZE before the aircraft arrives at Buenos Aires.

[3] Spraying is required EZE - SCL before arriving in Santiago, Chile.

TABLE S2. WHO-recommended aircraft cabin disinsection procedures

Methods	Insecticide	Applied by whom	Applied when	Additional comments	Air conditioning
**Pre-embarkation cabin treatment**	Permethrin 2% aerosol at a rate of 35 g / 100 m^3	Ground staff.	Before embarkation of passengers, at the departing airport.	Should be performed in conjunction with cargo hold disinsection if cargo holds were not previously treated with residual spray.	Must be turned off during application of spray and for 5 min after completion of spraying. Recirculation fans may be left on if essential for aircraft operation, but at lowest flow rate.
**Pre-departure method**	Aerosol of d-phenothrin 2% or 1R-trans-phenothrin 2% at a rate of 35 g / 100 m^3	Crew members.	After passenger embarkation but before the overhead lockers are closed and the aircraft is pushed back for departure.	Should be performed in conjunction with cargo hold disinsection if cargo holds were not previously treated with residual spray. All areas of the aircraft cabin are sprayed, including flight deck, open overhead and coat lockers and toilets.	During disinsection and for 5 min after completion of spraying, the aircraft's air-conditioning should be set off or to normal flow, and recirculation fans must be on.
**Pre-departure cargo hold disinsection**	Single-shot aerosol can with a vertical ejection nozzle containing permethrin 2% and d-phenothrin 2% (or 1R-trans-phenothrin 2%) or an aerosol containing d-phenothrin 2% or 1R-trans-phenothrin 2% at a rate	Ground staff.	Occurs at last departure airport after all cargo has been loaded and just before cargo hold door is closed. If small animals are to be loaded, should occur before animals are loaded but after all other cargo is on board.	Only applies if holds were not previously treated with residual spray.	Must remain off during disinsection and for 5 min after completion of spraying; recirculation fans may be left on if essential but should be set to the lowest rate

	of 35 g / 100 m^3				
**On-arrival disinsection method**	Aerosol of d-phenothrin 2% or 1R-trans-phenothrin 2% at a rate of 35 g / 100 m^3	Crew members.	On arrival.	To be conducted if airline has not conducted one of the approved pre-arrival procedures, authorities at arrival airport are not satisfied that operator has used the chosen method correctly, or additional on-arrival treatment is required by authorities at arrival airport. All galleys, toilets, lockers, crew rest areas, and flight deck to be sprayed with a 5-min saturation period to be observed.	Must remain off during disinsection and for 5 min after completion of spraying; recirculation fans may be left on if essential but should be set to the lowest rate
**Residual treatment**	Permethrin 2% EC at a rate of 0.2 g a.i./m^2 for internal surfaces, 0.5 g a.i./m^2 on floors	Trained, professional pest-control operators.	Every 8 weeks, when there are no passengers on board.	Residual treatment of the cabin should be performed in conjunction with cargo hold disinsection.	Must be turned off during treatment, but system and recirculation fans must be reactivated and run for at least 1 hour or in accordance with a.i. label guidance before passengers can embark the aircraft.

TABLE S3. Disinsection Regulations for Marine/Submarine Conveyances

General Ship Regulations		
All vessels must obtain a Ship Sanitation Control Certificate or a Ship Sanitation Control Exception Certificate every six months, as stated by the International Heath Regulations. This certificate should include the presence of any pest or insect as well as the method used to eradicate them from the vessel. If the certificate has expired or they do not have one, the ship will undergo inspection and further disinsection in places specifically where vectors (such as mosquitoes, cockroaches, etc.) fester as outlined by the WHO [5].		
**Specific regulations**		
Conveyance type	Country	Regulation
Ship	China	Yellow fever prevalent area including Nigeria, Peru, and Brazil ((except for Ceará, North Rio Grande, Texas, Paraiba, Pernambuco, Alagoas, Sergipe) [6].
	Republic of Korea	Zika affected countries; Certificate must indicate used of pyrethroid ingredient in insecticide used for disinsection. Disinsection must occur at least 1 hour prior to departure from affected countries [7].
	European Union (EU)*	Ports in the European Union Member States (EUMS) to disinsect the imported goods at the port facilities [8].

*__EU Countries include__ Austria, Belgium, Bulgaria, Croatia, Republic of Cyprus, Czech Republic, Denmark, Estonia, Finland, France, Germany, Greece, Hungary, Ireland, Italy, Latvia, Lithuania, Luxembourg, Malta, Netherlands, Poland, Portugal, Romania, Slovakia, Slovenia, Spain and Sweden.

Supplementary Table REFERENCES:

[1] *Aircraft Disinsection Requirements | US Department of Transportation*. https://www.transportation.gov/airconsumer/spray.

[2] Reviewing the topic of aircraft disinsection. Association of Canadian Travel Agencies and Travel Advisors. (n.d.). https://www.acta.ca/news-releases/cb0206

[3] Air Canada - health and travel tips. Air Canada. (n.d.). https://www.aircanada.com/ca/en/aco/home/plan/peace-of-mind/travel-tips.html#/

[4] Subiakto, Yuli. "Aviation Medicine Capacity on Facing Biological Threat In Indonesia Airports." *Infectious Disease Reports*, vol. 12, no. Suppl 1, July 2020, p. 8738. *PubMed Central*, https://doi.org/10.4081/idr.2020.8738.

[5] Canada, Health. *ARCHIVED - Ship Sanitation Certificate Program*. 4 Feb. 2009, https://www.canada.ca/en/health-canada/services/healthy-living/travel-health/general-advice/ship-sanitation-certificate-program-health-canada.html.

[6] *China: Yellow Fever Prevention Measures - The Swedish Club*. 12 Feb. 2018, https://www.swedishclub.com/news/loss-prevention/china-yellow-fever-prevention-measures/.

[7] *Zika Virus: South Korea Requires Self-Disinfection Certificates for Vessels - The Swedish Club*. 24 Mar. 2016, https://www.swedishclub.com/news/loss-prevention/zika-virus-south-korea-requires-self-disinfection-certificates-for-vessels/.

[8] Interim guidance on maritime transport and zika virus disease. (n.d.). https://www.shipsan.eu/Portals/0/docs/MaritimeZika_EUSHIPSAN_UPDATE_13.4.2016.pdf

Efficacy of Aircraft Disinsection Checklist

Section	Topic	No	Item	Adherence? (y/n)
1. Studies of Aerosol Disinsection of Passenger Cabins	Recommended Placement of Cage:	1	Place cages in three areas of the main passenger cabin: middle row of seats, fifth row from the front, and fifth row from the rear.	
		2	In each area, place six cages (three on the left and three on the right side) in specific locations: foot space beneath the window seat, bottom cushion of the aisle seat, and middle of the open overhead luggage compartment.	
		3	Additional cages in at least one toilet area and one galley area.	
	Recommended Number of Mosquitoes Tested:	1	Use 25 non-blood-fed, 2–5-day-old female mosquitoes per cage.	
	Recommended Number of Experimental Replicates:	1	Conduct a minimum of two replicates with susceptible mosquito species.	
		2	Test on both single- and dual-aisle seating aircraft.	
	Environmental Conditions for Testing:	1	Air-conditioning should be switched off during spraying and outer door closed.	
		2	Maintain a temperature of 23°C ± 2°C during the test.	
	Assessment Methods:	1	Assess knock-down at 60 minutes and mortality after 24 hours.	
2. Studies of Aerosol Disinsection of Cargo Holds	Recommended Placement of Cages:	1	Place four cages on the floor near each corner of the cargo hold; and three cages evenly spaced along the central axis of the hold at a height of 1 meter.	
		2	Hang four additional cages 10 cm from the ceiling and walls in each corner and ensure the central axis placement of the cages is at a height of 1 meter.	
	Recommended Number of Mosquitoes Tested:	1	Use 25 non-blood-fed, 2–5-day-old female mosquitoes per cage.	

	Recommended Number of Experimental Replicates:	1	Conduct at least two replicates.	
	Environmental Conditions for Testing:	1	Use ambient climatic conditions.	
	Assessment Methods:	1	Assess knock-down at 60 minutes and mortality after 24 hours.	
3. Studies of Long-term Residual Activity in Passenger Cabins	Recommended Placement of Cages:	1	Evaluate with a minimum of three representative internal aircraft surfaces (e.g., AerFilm®, carpet, curtains, wall panels).	
	Recommended Number of Mosquitoes Tested:	1	Use 10 non-blood-fed, susceptible female mosquitoes aged 2–5 days per WHO cone.	
	Recommended Number of Experimental Replicates:	1	Conduct three replicates from separately reared batches per location.	
	Environmental Conditions for Testing:	1	Maintain 27°C ± 2°C and 80% ± 10% relative humidity.	
	Assessment Methods:	1	Conduct WHO cone bioassays 24 hours after spraying and then at regular intervals (e.g., weekly) until mortality drops below 80%.	
4. Timing of Disinsection	Pre-flight Spraying:	1	Apply aerosol by ground staff before passengers board, not more than 1 hour before doors are closed.	
		2	Use aerosol containing insecticide with rapid action and limited residual action.	

Efficacy of Aircraft Disinsection Checklist

	Blocks-away:	1	Spray conducted by crew after passengers board and before take-off.	
		2	Air-conditioning should be switched off during spraying.	
	Top-of-descent:	1	Apply aerosol as the aircraft starts its descent.	
		2	Use aerosol containing insecticide for rapid action.	
	Residual Application:	1	Apply by professional pest control operators for long-term residual activity on aircraft interior surfaces.	
		2	Evaluate residual surface treatments weekly until mortality drops below 80%.	
5. General Guidelines	Cage Specifications:	1	Dimensions: Cylindrical steel-frame cages with a diameter of 90 mm and height of 150 mm.	
		2	Material: Nylon or polyester mesh netting with hole openings of 1.2 x 1.2 mm to 1.6 x 1.6 mm.	
		3	Placement: Cages should be positioned properly labeled (e.g., position of exposure, date of test) to ensure traceability and accurate interpretation of results.	
	Positive Control Inclusion:	1	Include a positive control (e.g., permethrin and D-phenothrin) in laboratory tests.	
	Mortality Thresholds for Control Groups:	1	If mortality in the control group exceeds 20%, the test is rejected.	
		2	If mortality in the control group is 0–20%, use Abbott's formula to correct the results with treated samples.	
	Data Analysis:	1	Analyze dose-response relations and determine the lethal dosage (LD50, LD90) using log-dose probit regression.	
		2	Report results with confidence intervals and correct mortality rates using Abbott's formula if necessary.	
6. Cited Rational for Non-Adherence				
